# Cryotolerance of brown bear (*Ursus arctos*) sperm depends on sperm origin: insights into sperm quality and proteomic profiles

**DOI:** 10.1038/s41598-026-43295-0

**Published:** 2026-05-08

**Authors:** Marta Neila-Montero, Luis Anel-Lopez, Rafael Montes-Garrido, Cristina Palacin-Martinez, Victoria Diez-Zavala, David Ramírez-González, Santiago Borragán, Patricia Manrique-Revuelta, Luis Anel, Mercedes Alvarez

**Affiliations:** 1 Investigación en Sanidad y Biotecnología (SaBio), IREC, CSIC - UCLM - JCCM, Albacete, 02006 España; 2 Genética, Departamento de Ciencia y Tecnología Agroforestal y Genética, Universidad de Castilla-La Mancha, Albacete, 02071 España; 3 Investigación en Técnicas de Reproducción Asistida (Itra-ULE), INDEGSAL, Universidad de León, León, 24071 España; 4 Reproducción Animal y Obstetricia, Departamento de Medicina, Cirugía y Anatomía Veterinaria, Universidad de León, León, 24071 España; 5 Anatomía, Departamento de Medicina, Cirugía y Anatomía Veterinaria, Universidad de León, León, 24071 España; 6 Parque de la Naturaleza de Cabárceno, Obregón, 39693 España; 7 Universidad de León (UNILEON), Área de Reproducción Animal y Obstetricia, Campus de Vegazana s/n, León, 24071 España

**Keywords:** Cryopreservation, Electroejaculation, Epididymis, Urethral catheterization, Semen, Sperm proteome, Biochemistry, Cell biology, Physiology, Zoology

## Abstract

Cryopreservation is a crucial tool for the *ex situ* conservation of endangered species, enabling the long-term storage of genetic resources. However, sperm from wild species often show variable cryoresistance, and the underlying molecular mechanisms remain poorly understood. This study evaluated how sperm origin (epididymal, pre-ejaculated, and ejaculated) influences the cryotolerance of brown bear (*Ursus arctos*) sperm by combining functional assessments (motility, viability, apoptosis, mitochondrial ROS production, and redox balance) with proteomic profiling before and after freezing. Epididymal sperm, despite superior initial motility, exhibited the greatest post-thaw decline in viability, apoptotic resistance, and mitochondrial ROS production. Ejaculated sperm showed intermediate pre-freeze values but retained the highest post-thaw quality, demonstrating better resilience to cryoinjury. Pre-ejaculated sperm, although starting with the lowest motility, displayed a more moderate decline, with intermediate post-thaw apoptosis and mitochondrial ROS levels. Redox analyses indicated higher basal oxidative stress in epididymal sperm and post-thaw reduction in antioxidant reserves in ejaculated sperm. Proteomic analyses identified 56, 33, and 53 downregulated proteins in epididymal, pre-ejaculated, and ejaculated sperm, respectively, with a core set of 22 proteins consistently affected across all sperm origins. Functional enrichment revealed that these shared proteins were mainly associated with energy and carbohydrate metabolism, highlighting core metabolic functions as common targets of cryodamage. In origin-specific analyses, epididymal sperm were the most affected, with disruptions in energy production, stress response, proteostasis, and fertilization processes. Pre-ejaculated sperm exhibited changes mainly in carbohydrate metabolism, while ejaculated sperm, although also affected in energy and stress processes, retained greater molecular stability, likely buffered by seminal plasma. These findings demonstrate that sperm origin strongly influences cryodamage patterns in the brown bear and identify a conserved metabolic signature of cryoinjury, supporting the value of proteomics for optimizing wildlife cryobanking strategies.

**Supplementary Information:**

The online version contains supplementary material available at 10.1038/s41598-026-43295-0.

## Introduction

With a broad circumpolar distribution, the brown bear (*Ursus arctos*) is currently classified as a species of least concern by the International Union for Conservation of Nature (IUCN), reflecting the stability and growth of several populations across northern Eurasia and North America^1^. However, this global status contrasts sharply with the situation in certain European regions, where small and isolated populations remain critically endangered^2^. In the Iberian Peninsula, the Cantabrian brown bear is the only surviving native population, occupying a fragmented range in the Cantabrian mountains of northern Spain^3^. Molecular evidence indicates that it may represent one of the last genetically unadmixed lineages of brown bears in Europe, reinforcing its unique conservation value^4^. Although recent recovery efforts have led to a moderate increase in population size –currently estimated at around 350 individuals divided into two subpopulations– this group remains highly vulnerable due to habitat fragmentation, human-wildlife conflict, and restricted gene flow, which exacerbate the risk of inbreeding and genetic erosion^5^.

To support the long-term viability of such vulnerable populations, *ex situ* conservation strategies have become increasingly important. Among them, sperm cryopreservation represents a promising tool for establishing genetic resource banks, allowing gametes to be stored indefinitely and later used in assisted reproductive technologies^3^. These biobanks serve as a safeguard against demographic or environmental crises and contribute to the genetic management of small or bottlenecked populations. Nevertheless, the successful implementation of sperm cryobanking in wild species, such as the brown bear, is hindered by the lack of species-specific protocols for sperm collection, processing, and freezing^6^. Most procedures are extrapolated from domestic species and often fail to accommodate the physiological and reproductive peculiarities of wild species^7^.

Despite its potential, sperm cryopreservation still presents several biological limitations. The freeze-thaw process imposes substantial physical and biochemical stress on sperm, leading to a range of structural and functional impairments across various species^8^. These cryoinjuries typically include plasma membrane destabilization, mitochondrial dysfunction, enhanced reactive oxygen species (ROS) generation, DNA damage, and activation of apoptotic-like pathways^9–14^. Consequently, frozen-thawed sperm often show reduced motility, viability, and fertilization ability^15^. Understanding the nature and extent of this cryoinjury is essential for improving cryopreservation strategies and ensuring high post-thaw sperm quality in conservation contexts.

Traditionally, sperm quality assessment relies on basic parameters, including motility, viability, and morphology^16^. However, these markers alone are often insufficient to predict post-thaw functionality. More advanced methods now assess structural integrity, mitochondrial activity, caspase activation, and redox status, providing a more comprehensive view of sperm health and function^17^. Importantly, cryopreservation may affect sperm differently depending on their initial status, and pre-freeze quality does not always predict post-thaw performance^18^. This highlights the need to evaluate samples before and after freezing to assess true cryotolerance.

In parallel, sperm proteomics has emerged as a powerful approach for investigating the molecular mechanisms underlying cryoresistance and sperm functionality after freeze-thawing^19^. Several studies have demonstrated that specific protein families, particularly those involved in energy metabolism (glycolysis, citric acid cycle, and oxidative phosphorylation), motility, protein folding, and oxidative stress response, are consistently altered in sperm after cryopreservation^20–23^. These findings suggest that proteomic profiling could serve as a predictive tool for sperm freezability. However, while these approaches have yielded valuable insights into domestic animals^24^ and some wild species, such as mountain small ruminants^25^, buffalo^26^, dromedary camels^27^, and sharks^28^, no studies to date have explored the proteomic impact of cryopreservation in any ursid species. Our research group has previously demonstrated significant variation in both sperm quality and proteomic composition depending on the origin of the sperm in the brown bear^29^. Nevertheless, how each sperm origin responds to freezing, and whether their molecular response differ, remain unknown.

Thus, this study investigated how brown bear (*Ursus arctos*) sperm from three origins (epididymal, pre-ejaculated, and ejaculated) respond to cryopreservation by comparing quality parameters and proteomic features before and after freezing. By integrating functional and proteomic data, we aim to elucidate the biological processes underlying origin-dependent responses to cryopreservation and to optimize sperm cryobanking protocols for this species of high conservation concern.

## Materials and methods

### Ethical approval

All procedures involving brown bears (*Ursus arctos*) complied with relevant Spanish and European regulations, including RD 1386/2018 and Directive 2010/63/EU on the protection of animals used for scientific purposes.

The Animal Care and Use Committee of the University of León reviewed and approved the protocol under approval number ETICA-ULE-031-2023.

Authorization for animal research was granted to the Itra-ULE research group through a cooperative framework agreement between the University of León and Cantur S.A., the company responsible for managing the Parque de la Naturaleza de Cabárceno. Sample collection was performed exclusively during scheduled veterinary interventions routinely carried out in the park, including health monitoring procedures, population management actions, and general welfare assessments. All immobilization, handling, and sampling procedures were conducted by or under the supervision of qualified personnel from the park, so no additional permits from regional environmental authorities were required.

Veterinarians followed best-practice standards for wildlife anesthesia during chemical immobilization.

This study is reported in accordance with the ARRIVE guidelines.

### Animals

The experiments involved 14 adult *Ursus arctos* males sampled during the breeding season (late April-early July) across three consecutive years (2022-2024). These bears lived in semi-free conditions at Parque de la Naturaleza de Cabárceno in northern Spain (43° 21′ N, 3° 50′ W; 143 m altitude) and fed mainly on chicken, bread, and fruit.

Pre-ejaculated and ejaculated samples were obtained during the same immobilization session, whereas epididymal samples were recovered from the same bears during an independent veterinary procedure linked to population-control activities.

### Sperm collection

Sperm collection procedures were performed according to protocols previously described and validated in brown bears by our research group^29^. Remote drug delivery was used to anesthetize the males, employing a combination of 750 mg zolazepam HCl-tiletamine HCl (Zoletil^®^ 100; Virbac, Carros, France) and 6 mg medetomidine (Zalopin^®^ 10; Orion Pharma Animal Health, Finland). Throughout the anesthetic period, each animal was weighed, and physiological parameters (heart rate, respiratory rhythm, and peripheral oxygen saturation) were tracked continuously.

### Epididymal sperm

The scrotal area of 9 brown bears was shaved, cleansed, and disinfected before castration or epididymectomy. The excised epididymides were carefully dissected, removing surrounding connective tissue and superficial blood vessels to minimize contamination. Sperm were recovered by making multiple incisions in the cauda epididymis with a sterile scalpel and collecting emerging fluid in 0.5 mL graduated microtubes (*n* = 9).

### Pre-ejaculated and ejaculated sperm

The genital area of 14 males was prepared by cleaning the skin and rinsing the penis with sterile saline solution, followed by manual evacuation of the rectum. To avoid urine contamination of sperm samples, bladder catheterization was carried out before electroejaculation. In some individuals (*n* = 9), this step enabled recovery of a pre-ejaculatory sperm fraction from the penile urethra via catheterization, prior to any electroejaculatory stimulation.

A PT Electronics electroejaculator (Boring, OR, USA) with a 320 mm in length and 26 mm in diameter rectal probe was then used to obtain ejaculates. Stimulation pulses averaging 10 V and 250 mA were applied until ejaculation occurred (*n* = 14). Seminal fractions were collected separately in 15 mL glass tubes with graduations.

Immediately after sample collection, ejaculate volume was determined using the tube graduations, osmolarity was analyzed with a cryoscopic osmometer (OSMOMAT 3000; Gonotec, Berlin, Germany), sperm concentration was quantified using an automated cell counter (NucleoCounter SP-100; ChemoMetec, Allerod, Denmark), and motility was subjectively evaluated under phase-contrast microscopy at 100× magnification (Eclipse E400; Nikon, Tokyo, Japan). Fractions of comparable quality obtained during the same electroejaculation session were pooled to form a single ejaculate.

Samples (both pre-ejaculated and ejaculated) with low sperm concentration (< 150 × 10^6^ sperm/mL) were centrifuged at 600 × *g* for 6 min at room temperature, and the resulting pellets were processed to continue the experiment.

### Sperm processing (dilution, freezing and thawing)

Sperm samples from the three origins (epididymal, pre-ejaculated, and ejaculated) were first mixed 1:1 (v/v) with a TES-Tris-Fructose (TTF) medium prepared according to Anel *et al.*^30^. Briefly, the extender was based on a TES-Tris buffer stock solution prepared in Milli-Q water (TES 74.5 g/L; Tris 39.4 g/L) adjusted to 300 mOsm/kg and pH 7.2. The final extender was composed of 67% TES-Tris buffer, 4% fructose (from a 58.6 g/L stock solution in Milli-Q water), 20% clarified egg yolk, 6% glycerol, 2% EDTA, and 1% Equex paste (Minitüb, Tiefenbach, Germany)^30^. Tubes containing the diluted samples were placed in water at room temperature and transferred to a portable refrigerator (CoolFreeze CF-25; Dometic Group, Stockholm, Sweden), allowing the sperm samples to gradually cool to 5 °C over 70-80 min. Afterwards, the 3% glycerol samples were diluted again 1:1 (v/v) using a TTF diluent containing 9% glycerol (64% TES-Tris buffer, 4% fructose, 20% clarified egg yolk, 9% glycerol, 2% EDTA, and 1% Equex paste) to achieve a final glycerol concentration of 6%. Further dilution with the original extender (6% glycerol) was performed to adjust the final sperm concentration to 100 × 10^6^ sperm/mL. After 4 h of equilibration at 5 °C, sperm functionality and proteome were evaluated before freezing (pre-freeze assessment).

Extended samples were loaded into 0.25 mL straws, each containing 25 × 10^6^ sperm. Cryopreservation was performed using a controlled-rate freezer (Planer Kryo-Series III; Planer PLC, Middlesex, UK), programmed to cool at a rate of −20 °C/min from 5 °C to −100 °C. Once frozen, straws were transferred and stored in liquid nitrogen (–196 °C) until assessment of thawed samples. Thawing was performed by immersing the straws in a water bath at 65 °C for 6 s. After equilibration at room temperature for 10 min, post-thaw sperm functionality and proteomic profile were evaluated (post-thaw assessment).

### Sperm functionality evaluation

#### Sperm motility and kinetic parameters

Sperm motility and kinematics were evaluated using a Computer-Assisted Sperm Analysis (CASA) system (Sperm Class Analyzer^®^ version 6.4.0.89; Microptic S.L., Barcelona, Spain), following the analytical approach previously described by Neila-Montero *et al.*^29^ for this species. Recordings were obtained at 100 frames/s over a total of 50 frames, and only particles with areas ranging from 12 to 62 μm² were included in the analysis.

Samples were adjusted to 25 × 10^6^ sperm/mL using a glycerol-free TTF-based extender composed of 95% TES-Tris buffer, 4% fructose, and 1% clarified egg yolk (320 mOsm/kg, pH 7.2) and equilibrated for 5 min on a warming plate at 37 °C. A 5 µL aliquot of each diluted sample was then loaded into a Makler chamber (10 μm depth; Sefi Medical Instruments, Haifa, Israel) and examined on a heated microscope stage (37 °C) at 100× magnification using phase-contrast microscopy. Image capture was performed with a BASLER acA1300-200uc digital camera (Basler Vision Technologies, Ahrensburg, Germany).

For each sample, a minimum of 400 sperm from four randomly chosen fields were evaluated, excluding non-sperm particles. The kinematic variables reported included curvilinear velocity (VCL, µm/s), linearity (LIN, %), and amplitude of lateral head displacement (ALH, µm). Total motility (TM), progressive motility (PM), and fast progressive motility (FPM) were calculated as the proportions of sperm exceeding VCL thresholds of 15, 45, and 75 μm/s, respectively.

#### Sperm viability, apoptosis, and mitochondrial ROS production

Aliquots of each sample containing 2 × 10^6^ sperm (20 µL, given an initial concentration of 100 × 10^6^ sperm/mL) were washed in 1 mL of phosphate-buffered saline (PBS; 300 mOsm/kg, pH 7.2) by a short 15-second centrifugation spin (MiniSpin plus; Eppendorf, Hamburg, Germany), after which the supernatant was removed. Multiparametric fluorescent staining was performed according to the workflow reported by Riesco *et al.*^31^. Each pellet was incubated for 30 min at room temperature and protected from light simultaneously with three fluorescent probes: Zombie Violet™ Fixable Viability Kit (1:1000 final dilution in PBS; BioLegend, San Diego, CA, USA) to assess plasma membrane integrity, CellEvent™ Caspase-3/7 Green Detection Reagent (4 µM final concentration in PBS; ThermoFisher, Waltham, MA, USA) to detect apoptosis, and CellROX™ Deep Red Reagent (5 µM final concentration in PBS; Invitrogen, Eugene, OR, USA) to evaluate mitochondrial ROS production. Following incubation, cells were rewashed to remove excess stain, and pellets were resuspended in 1 mL of PBS and immediately subjected to flow cytometry.

Flow-cytometric measurements were performed on a CytoFLEX S instrument (Beckman Coulter, Brea, CA, USA) equipped with four excitation lasers (405, 488, 561, and 638 nm). Sperm cells were initially identified based on their characteristic forward scatter (FSC) and side scatter (SSC) properties, allowing exclusion of debris and non-sperm particles. Events within this gate were further refined by selecting single cells using SSC-A vs. SSC-H plots. For each sample, 40,000 total events were collected at an acquisition rate of 200-300 events/s, ensuring that at least 20,000 gated sperm were included. Fluorescence signals from Zombie Violet™, CellEvent™ Caspase-3/7 Green, and CellROX ™ Deep Red were detected in FL5 (excitation 405 nm, emission 450/45 band pass (BP) filter), FL1 (excitation 488 nm, emission 525/40 BP), and FL4 (excitation 638 nm, emission 660/20 BP), respectively. Data were processed with FlowJo™ software (version 10.8.1; Ashland, Wilmington, DE, USA). Within the gated sperm singlet population, viable sperm were identified by low Zombie Violet™ signal (intact membrane), apoptotic sperm by positivity for CellEvent™ Caspase-3/7 Green (active caspases 3 and 7), and sperm exhibiting high mitochondrial ROS content by strong CellROX™ Deep Red fluorescence (high superoxide production).

#### Sperm oxidation-reduction potential

The redox balance of the sperm samples was assessed using the RedoxSYS™ diagnostic system (Luoxis Diagnostics, Englewood, CO, USA). For each measurement, 1 × 10^6^ sperm cells were washed with 100 µL of PBS and subsequently resuspended in 20 µL of the same buffer. The suspension was then dispensed onto a single-use sensor, which was inserted into the device. Within 4 min, the system generated two outputs: the static oxidation-reduction potential (sORP) in millivolts (mV), which reflects the overall balance between oxidants and reductants; and the capacitance oxidation-reduction potential (cORP) in microcoulombs (µC), which represents the antioxidant reserve capacity of the sample.

### Sperm proteome evaluation

Sperm samples (200 × 10^6^ sperm) diluted in TTF-based extender were centrifuged at 10,000 × *g* for 15 min at 4 °C, and the resulting pellets were stored at −80 °C until analysis. Sample purity was confirmed by phase-contrast microscopy. Protein extraction, quantification, and enzymatic digestion were performed as described by Martín-Cano *et al.*^32^. In brief, each sperm pellet was resuspended in 400 µL of Protein Extraction Reagent Type 4 (7.0 M urea, 2.0 M thiourea, 40 mM Trizma^®^ base, and 1.0% C7BzO; pH 10.4) and incubated for 1 h at 4 °C under continuous rotation. The lysates were then centrifuged at 17,000 × *g* for 30 min at room temperature to remove insoluble material, and the clarified supernatant was collected. Protein levels were determined using the 2-D Quant Kit (GE Healthcare, Sevilla, Spain), and 100 µg of protein from each sample were reduced with dithiothreitol (DTT), alkylated with iodoacetamide (IAA), and subjected to in-solution tryptic digestion at a protein: trypsin ratio of 100:1 (w/w) for a minimum of 3 h and up to overnight at 37 °C, following the referenced protocol.

Proteomic profiling was carried out in technical duplicate whenever sample availability allowed, using a UHPLC-Q-TOF platform (Agilent Technologies, Santa Clara, CA, USA), consisting of a 1290 Infinity II UHPLC system coupled to a 6550 Q-TOF mass spectrometer equipped with an Agilent Jet Stream Dual ESI source. Data acquisition was managed through MassHunter Workstation software (Rev. B.06.01; Agilent Technologies, Santa Clara, CA, USA). Peptide mixtures (75 µg) were resolved on an AdvanceBio Peptide Mapping column (2.7 μm, 150 × 2.1 mm; Agilent Technologies, Santa Clara, CA, USA) maintained at 55 °C with a flow rate of 0.4 mL/min. Chromatographic separation gradient began with 2% buffer B (acetonitrile/water/formic acid, 89.9:10:0.1) for 5 min, increased linearly to 45% in 40 min, then to 95% in 15 min, and was held for 5 min. Finally, it was re-equilibrated for 5 min.

Mass spectrometry was performed in positive ion mode under the following conditions: nebulizer pressure 35 psi, drying gas 10 L/min at 250 °C, sheath gas 12 L/min at 300 °C, capillary voltage 3,500 V, fragmentor 340 V, and octopole RF 750 V. MS and MS/MS spectra were acquired in extended dynamic range mode (50-1,700 *m/z*) at 8 and 3 spectra/s, respectively. Auto MS/MS acquisition selected up to 20 precursors per cycle based on abundance, using a ramped collision energy (slope of 3.6, offset −4.8). Previously fragmented ions were excluded after two consecutive scans.

Raw data were processed using the Spectrum Mill MS Proteomics Workbench (Rev. B.04.01; Agilent Technologies, Santa Clara, CA, USA). Parameters included precursor mass range 50-10,000 *m/z*, charge states up to +5, retention time/mass tolerances ± 60 s, signal-to-noise ratio (S/N) ≥ 25, and monoisotopic mass selection. MS/MS spectra were searched against the UniProt/Bear protein database (downloaded in October 2024) with tryptic digestion (≤ 5 missed cleavages), variable modifications (including carbamidomethylated cysteines), a peptide mass tolerance of 20 ppm, and a fragment mass tolerance of 50 ppm. A target False Discovery Rate (FDR) of 1.2% was applied for peptide validation using auto-thresholding, followed by protein-level polishing with an FDR set at 0%, meaning that only proteins supported by peptides passing the peptide-level FDR threshold were retained, without allowing additional protein-level false positives.

### Bioinformatic analysis of proteomic data

All bioinformatic analyses were conducted using R software (version 4.1.2; Auckland, New Zealand). Protein abundance data were quantile-normalized before statistical analysis. Within each sperm origin (epididymal, pre-ejaculated, and ejaculated), differential protein enrichment between pre-freezing and post-thawing samples was assessed using linear models implemented in the limma package (version 3.50.3). *P*-values were adjusted for multiple testing using the Benjamini-Hochberg method.

The resulting list of significantly downregulated proteins for each sperm origin was independently queried against the Ursidae database in STRING (https://string-db.org/) to explore enriched biological processes (gene ontology) in brown bear sperm.

Heatmaps illustrating changes in protein abundance before and after cryopreservation within each sperm origin were generated using the heatmap3 package (version 1.1.9). Due to the high proportion of missing values (NA) observed for some proteins across samples, these heatmaps were constructed using only those differentially abundant proteins that were consistently quantified across all samples within each sperm origin. This approach ensured robust visualization of abundance patterns while the complete set of quantified and statistically evaluated proteins was retained for differential analysis and functional enrichment.

In addition, a dedicated heatmap summarizing the abundance patterns of the 22 proteins consistently downregulated across all sperm origins was generated using the ComplexHeatmap package. This visualization integrated all samples and sperm origins to provide an overview of the shared proteomic response to cryopreservation.

Due to the lack of a fully curated *Ursus arctos* proteome, protein identification was based on orthologous entries from related *Ursus* species available in public databases. Consequently, multiple UniProt entries corresponding to the same protein but annotated in different species (e.g., *Ursus americanus* or *Ursus maritimus*) may appear in the dataset. These entries were treated as independent protein features during identification and quantification, reflecting database structure and peptide-to-protein assignment rather than true biological differences in protein regulation.

### Statistical analysis

All statistical analyses were carried out using the SAS/STAT^®^ statistical package, version 9.4 (SAS Institute Inc., Cary, NC, USA). Graphs were created using Prism 10 (GraphPad Software, San Diego, CA, USA).

Two independent analyses were conducted: one based on absolute values, and another based on differential rates, calculated as the ratio between post-thaw and pre-freeze values (DR = [post-thaw/pre-freeze] × 100) to measure the cryopreservation-induced decline in sperm quality while accounting for baseline differences among samples.

Both analyses were conducted using mixed linear models (MIXED procedure). For the analysis of absolute values, sample type (pre-freeze and post-thaw), sperm origin (epididymal, pre-ejaculated, ejaculated), and their interaction were included as fixed effects, while male identity (bear ID) was specified as a random effect. Comparisons among sperm origins were performed within each sample type, and comparisons between sample types were made within each sperm origin. In the differential rate analysis, sperm origin was included as a fixed effect, and bear ID was again considered a random effect. Post hoc comparisons were performed using Tukey’s test to adjust for multiple comparisons. Results are presented as mean ± standard error of the mean (SEM). Statistical significance was set at *p* < 0.05. The number of asterisks indicated the significance levels: one asterisk (*) for *p* < 0.05, two asterisks (**) for *p* < 0.01, three asterisks (***) for *p* < 0.001, and four asterisks (****) for *p* < 0.0001.

## Results

### Sperm motility and kinetic parameters

**Fig. 1:**
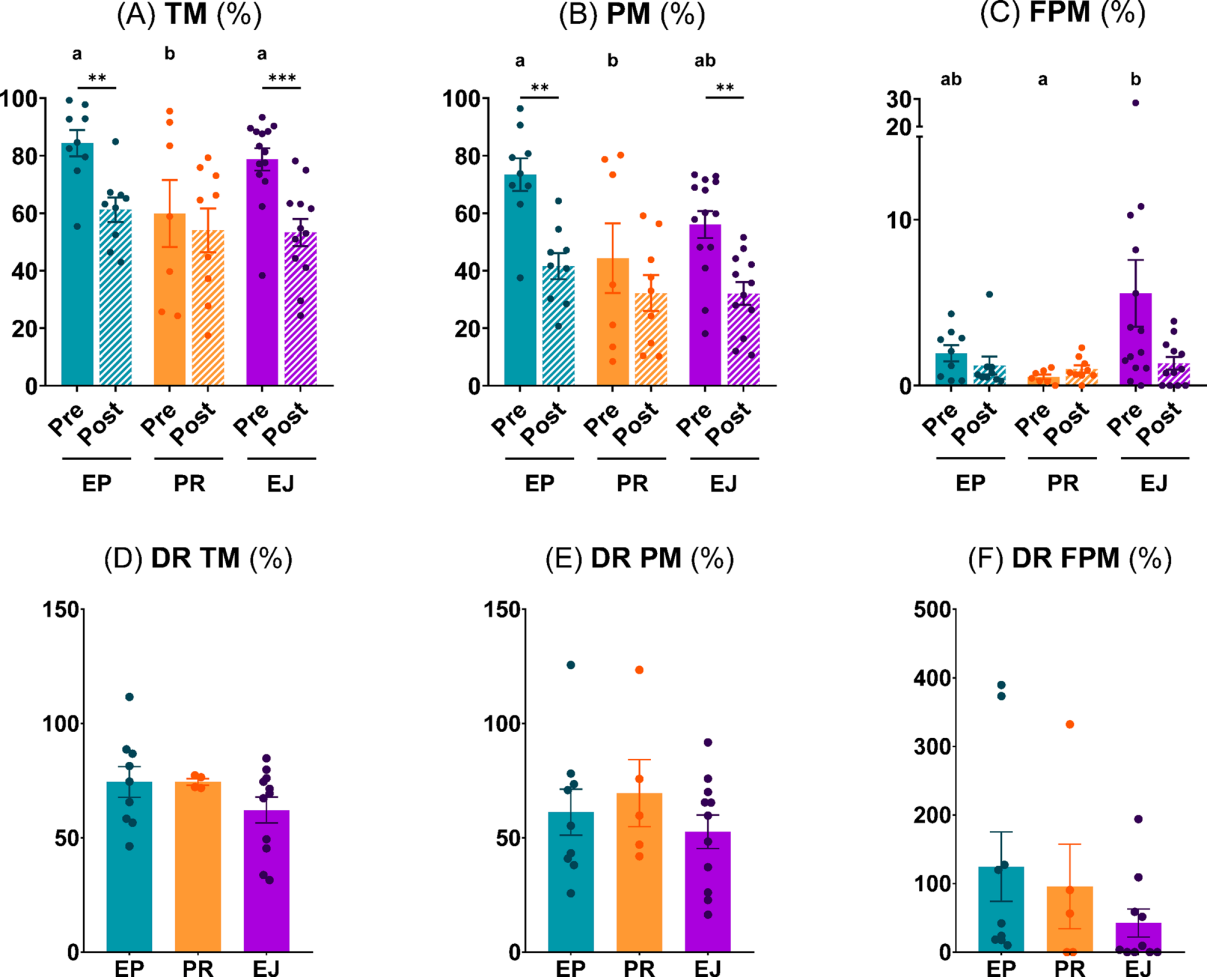
Brown bear sperm motility parameters in pre-freeze (Pre) and post-thaw (Post) samples, and differential rate (DR = post-thaw/pre-freeze × 100). (**A**) Total motility (TM, %); (**B**) Progressive motility (PM, %); (**C**) Fast progressive motility (FPM, %); (**D**) Differential rate of total motility (DR TM, %); (**E**) Differential rate of progressive motility (DR PM, %); (**F**) Differential rate of fast progressive motility (DR FPM, %). Each dot represents an individual male. Different lowercase superscripts (a, b) denote statistically significant variation (*p* < 0.05) among sperm origins (EP, epididymal; PR, pre-ejaculated; EJ, ejaculated) within pre-freeze samples. Non-significant differences (*p* ≥ 0.05) were observed among sperm origins within post-thaw samples. Asterisks (**, ***) indicate significant changes (*p* < 0.01 and *p* < 0.001, respectively) between pre-freeze and post-thaw values within each sperm origin. Comparisons of differential rates showed no statistically significant differences (*p* ≥ 0.05).

In post-thawed samples, no differences were observed among sperm origins for any motility parameters (TM, PM or FPM) (*p* ≥ 0.05) (Figs. 1A-C).

In contrast, in pre-freeze samples, pre-ejaculated sperm showed the lowest values for TM, PM, and FPM. Specifically, TM was significantly lower in pre-ejaculated samples compared to both epididymal and ejaculated sperm (*p* < 0.05) (Fig. 1A). PM was also significantly lower in pre-ejaculated sperm than in epididymal samples (*p* < 0.05), while ejaculated sperm showed intermediate values with non-significant differences from either of the other two origins (*p* ≥ 0.05) (Fig. 1B). For FPM, pre-ejaculated sperm exhibited significantly lower values than ejaculated sperm (*p* < 0.05), whereas epididymal sperm did not differ significantly from the other two groups (*p* ≥ 0.05) (Fig. 1C).

When comparing pre-freeze and post-thaw values within each sperm origin, significant reductions in TM and PM were detected in epididymal and ejaculated samples (*p* < 0.05), while no changes were observed in pre-ejaculated samples (*p* ≥ 0.05) (Fig. 1A and B).

Regarding differential rates (DR), non-significant differences were found among the three sperm origins for any of the motility parameters analyzed (*p* ≥ 0.05) (Figs. 1D-F).

**Fig. 2:**
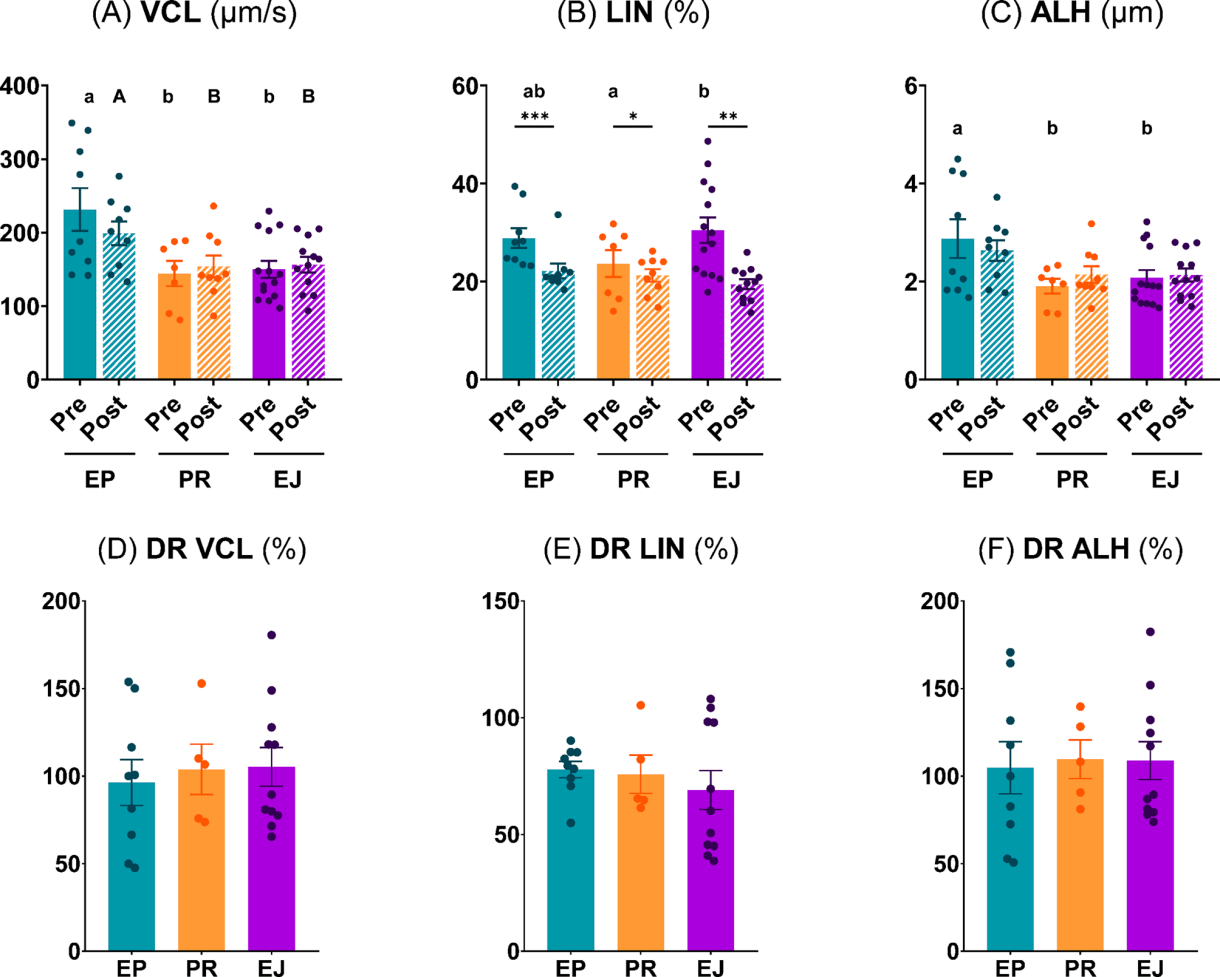
Brown bear sperm kinetic parameters in pre-freeze (Pre) and post-thaw (Post) samples, and differential rate (DR = post-thaw/pre-freeze × 100). (**A**) Curvilinear velocity (VCL, µm/s); (**B**) Linearity (LIN, %); (**C**) Amplitude of lateral head displacement (ALH, µm); (**D**) Differential rate of curvilinear velocity (DR VCL, %); (**E**) Differential rate of linearity (DR LIN, %); (**F**) Differential rate of amplitude of lateral head displacement (DR ALH, %). Each dot represents an individual male. Different lowercase superscripts (a, b) denote statistically significant variation (*p* < 0.05) among sperm origins (EP, epididymal; PR, pre-ejaculated; EJ, ejaculated) within pre-freeze samples. Different capital superscripts (A, B) denote statistically significant variation (*p* < 0.05) among sperm origins within post-thaw samples. Asterisks (*, **, ***) indicate significant changes (*p* < 0.05, *p* < 0.01, and *p* < 0.001, respectively) between pre-freeze and post-thaw values within each sperm origin. Comparisons of differential rates showed no statistically significant differences (*p* ≥ 0.05).

All kinetic parameters evaluated showed significant differences among sperm origins in pre-freeze samples. In particular, VCL and ALH were significantly higher in epididymal sperm compared to both pre-ejaculated and ejaculated sperm (*p* < 0.05) (Figs. 2A and C). LIN was significantly higher in ejaculated sperm than in pre-ejaculated sperm (*p* < 0.05), while epididymal sperm showed intermediate values with non-significant differences from either of the other two origins (*p* ≥ 0.05) (Fig. 2B).

In post-thaw samples, significant differences among sperm origins were observed only for VCL, which remained higher in epididymal sperm compared to pre-ejaculated and ejaculated sperm (*p* < 0.05) (Fig. 2A).

When comparing pre-freeze and post-thaw values within each sperm origin, significant reductions in LIN were detected in all three groups. The level of significance varied, being greatest for epididymal sperm (*p* < 0.001), followed by ejaculated sperm (*p* < 0.01), and pre-ejaculated sperm (*p* < 0.05) (Fig. 2B).

Regarding the DR, no differences were observed among sperm origins for any of the kinetic parameters analyzed (*p* ≥ 0.05) (Figs. 2D-F).

### Sperm viability, apoptosis, and mitochondrial ROS production

**Fig. 3:**
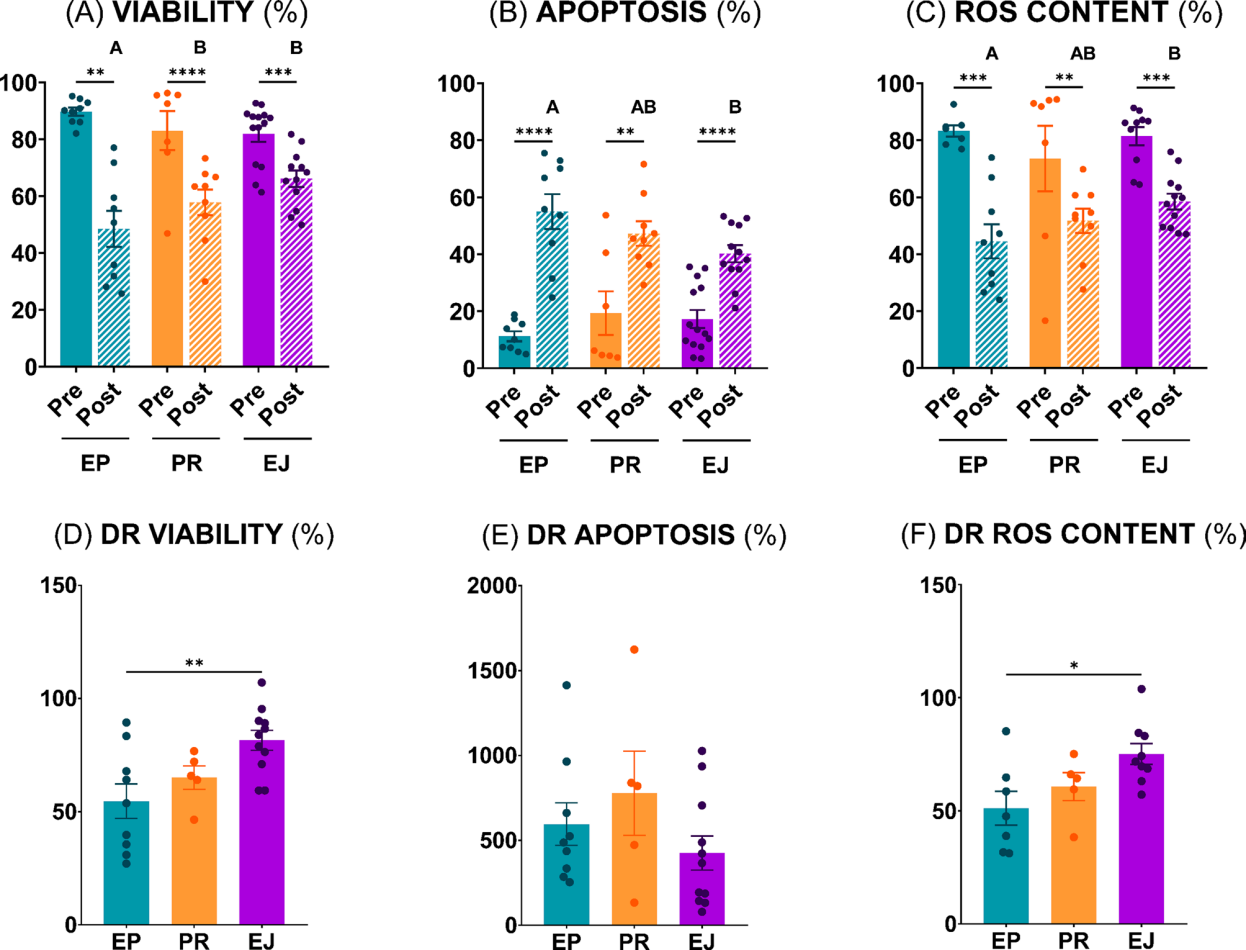
Brown bear sperm flow cytometry parameters in pre-freeze (Pre) and post-thaw (Post) samples, and differential rate (DR = post-thaw/pre-freeze × 100). (**A**) Viable sperm assessed with Zombie Violet™ (%); (**B**) Apoptotic sperm assessed with CellEvent™ Caspase-3/7 Green (%); (**C**) Sperm with high mitochondrial ROS content assessed through the superoxide production with CellROX™ Deep Red (%); (**D**) Differential rate of viable sperm (DR viability, %); (**E**) Differential rate of apoptotic sperm (DR apoptosis, %); (**F**) Differential rate of sperm with high mitochondrial ROS content (DR ROS content, %). Each dot represents an individual male. Non-significant differences (*p* ≥ 0.05) were observed among sperm origins (EP, epididymal; PR, pre-ejaculated; EJ, ejaculated) within pre-freeze samples. Different capital superscripts (A, B) denote statistically significant variation (*p* < 0.05) among sperm origins within post-thaw samples. Asterisks (*, **, ***, ****) indicate significant changes (*p* < 0.05, *p* < 0.01, *p* < 0.001, and *p* < 0.0001, respectively) between pre-freeze and post-thaw values within each sperm origin and among differential rates.

In pre-freeze samples, non-significant differences were observed among sperm origins for any of the flow cytometry parameters evaluated (viability, apoptosis, and mitochondrial ROS content as inferred from superoxide production) (*p* ≥ 0.05) (Figs. 3A-C).

In contrast, all three parameters showed significant differences among sperm origins in post-thaw samples. Viability was significantly lower in epididymal sperm compared to both pre-ejaculated and ejaculated sperm (*p* < 0.05) (Fig. 3A). Apoptosis levels were significantly lower and ROS content significantly higher in ejaculated sperm compared to epididymal sperm (*p* < 0.05), while pre-ejaculated sperm showed intermediate values without significant differences from the other two groups (*p* ≥ 0.05) (Figs. 3B and C).

When comparing pre-freeze and post-thaw values within each sperm origin, significant changes were observed for all parameters in all three sperm origins (*p* < 0.05). Both sperm viability and mitochondrial ROS production decreased after cryopreservation, while apoptosis levels increased, regardless of sperm origin (Figs. 3A-C).

For DR comparisons, significant differences were found between epididymal and ejaculated sperm for both viability and mitochondrial ROS production, with ejaculated sperm showing higher values in both cases (*p* < 0.05) (Figs. 3D and E). Non-significant differences were detected for apoptosis DR (*p* ≥ 0.05) (Fig. 3F).

### Sperm oxidation-reduction potential

**Fig. 4:**
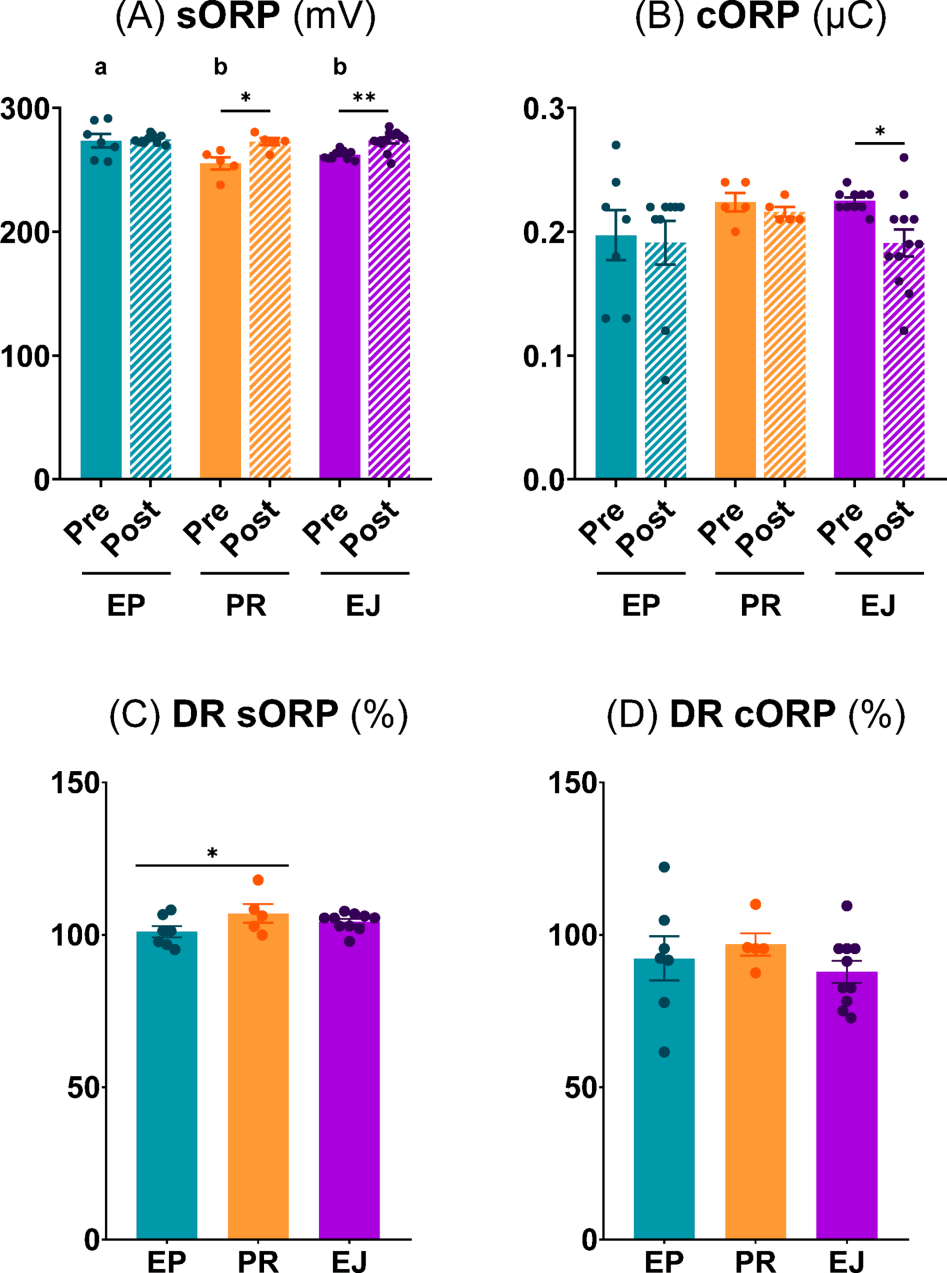
Brown bear sperm oxidation-reduction parameters in pre-freeze (Pre) and post-thaw (Post) samples, and differential rate (DR = post-thaw/pre-freeze × 100). (**A**) Static ORP index (sORP, mV); (**B**) Capacitance ORP index (cORP, µC); (**C**) Differential rate of static ORP index (DR sORP, %); (**D**) Differential rate of capacitance ORP index (DR cORP, %). Each dot represents an individual male. Different lowercase superscripts (a, b) denote statistically significant variation (*p* < 0.05) among sperm origins (EP, epididymal; PR, pre-ejaculated; EJ, ejaculated) within pre-freeze samples. Non-significant differences (*p* ≥ 0.05) were observed among sperm origins within post-thaw samples. Asterisks (*, **) indicate significant changes (*p* < 0.05 and *p* < 0.01, respectively) between pre-freeze and post-thaw values within each sperm origin and among differential rates.

In post-thaw samples, neither sORP nor cORP showed significant differences among sperm origins (*p* ≥ 0.05) (Figs. 4A and B).

In contrast, in pre-freeze samples, sORP was significantly higher in epididymal sperm compared to both pre-ejaculated and ejaculated sperm (*p* < 0.05) (Fig. 4A), while non-significant differences were found for cORP (*p* ≥ 0.05) (Fig. 4B).

When comparing values within each sperm origin before and after cryopreservation, they significantly increased after thawing in both pre-ejaculated and ejaculated sperm (*p* < 0.05) (Fig. 4A). In contrast, cORP showed a significant post-thaw reduction only in ejaculated sperm (*p* < 0.05), without changes in the other two origins (*p* ≥ 0.05) (Fig. 4B).

Regarding DR values, significant differences were observed only for sORP, with pre-ejaculated sperm showing higher values than epididymal sperm (*p* < 0.05) (Fig. 4C). Non-significant differences were found for cORP DR (*p* ≥ 0.05) (Fig. 4D).

### Sperm proteome

Across all sperm origins and experimental conditions, a total of 668 proteins were consistently quantified after quality control and normalization. Differential abundance analyses were then conducted within each sperm origin. The complete lists of quantified and differentially abundant proteins, including UniProt IDs, abundance values, fold changes, and statistical parameters, are provided as Supplementary Data S1-S3.

Although both up- and downregulated proteins were identified, the number of upregulated proteins after cryopreservation was very limited across all sperm origins (8, 3, and 3 for epididymal, pre-ejaculated, and ejaculated sperm, respectively). Therefore, subsequent result descriptions focused mainly on downregulated proteins, which constituted the predominant proteomic response to cryopreservation and enabled robust GO:BP enrichment analysis.

**Fig. 5:**
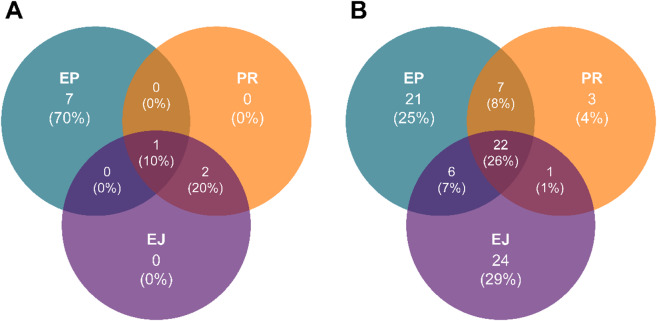
Venn diagrams showing the overlap of significantly (**A**) upregulated and (**B**) downregulated proteins after cryopreservation across sperm origins. Proteins were considered differentially abundant using *q* < 0.05 and |log₂ fold change| ≥ 1 (post-thaw vs. pre-freeze, within each sperm origin –EP, epididymal; PR, pre-ejaculated; EJ, ejaculated–). Numbers indicate the count of proteins per region, and percentages are relative to the total number of upregulated or downregulated proteins across the three origins.

An overlap analysis of upregulated proteins (*q* < 0.05; |log₂ fold change| ≥ 1) across sperm origins revealed a very limited shared response. Only one protein –A0A452Q891, corresponding to Sodium/potassium-transporting ATPase subunit alpha, American black bear (*Ursus americanus*)– was consistently upregulated in all three origins, whereas the remaining upregulated proteins showed mostly origin-specific patterns (Fig. 5A).

In contrast, the overlap analysis of downregulated proteins (*q* < 0.05; |log₂ fold change| ≥ 1) showed both shared and origin-specific responses to cryopreservation. A core set of 22 proteins (26%) was consistently downregulated in all three origins, whereas 21 (25%), 3 (4%), and 24 (29%) proteins were uniquely downregulated in epididymal, pre-ejaculated, and ejaculated sperm, respectively. Pairwise overlaps comprised 7 proteins (EP-PR), 6 (EP-EJ), and 1 (PR-EJ) (Fig. 5B).

The complete list of the 22 proteins consistently downregulated across all sperm origins, including UniProt IDs, protein names, and annotated species, is provided in Table S1.

**Fig. 6:**
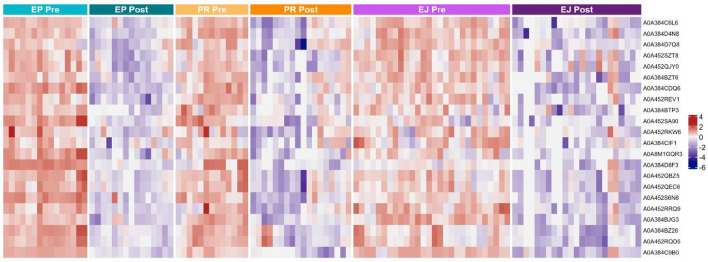
Heatmap showing the abundance patterns of the 22 proteins consistently downregulated after cryopreservation across all sperm origins in brown bear sperm. Proteins are clustered using hierarchical clustering. Each row represents an individual protein (labelled with its UniProt ID), and each column corresponds to an individual proteomic run (technical replicate) from epididymal (EP), pre-ejaculated (PR), and ejaculated (EJ) sperm samples (Pre, pre-freeze; Post, post-thaw). The color scale indicates normalized expression levels, with red areas representing higher and blue areas representing lower relative protein abundance. Proteins were normalized and filtered by a |log₂ fold change| ≥ 1 and *q* < 0.05.

To further characterize this core set of 22 proteins, their abundance patterns were visualized using a heatmap including all samples (Fig. 6). Despite the different sperm sources, these proteins showed a highly consistent decrease after thawing, indicating that cryopreservation was the main factor driving their differential abundance rather than sperm origin.

**Fig. 7:**
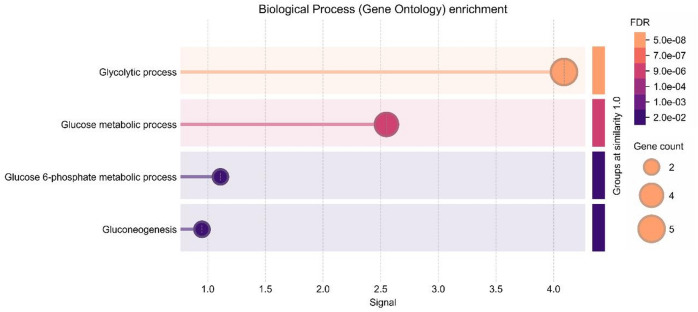
GO:BP enrichment analysis of the 22 proteins consistently downregulated after cryopreservation across all sperm origins in brown bear sperm. Enriched biological processes are ranked by signal strength to emphasize those with the strongest functional relevance. The circle diameter corresponds to the number of proteins mapped to each process, and the color scale denotes the false discovery rate (FDR), with orange indicating more significant enrichments (lower FDR) and purple indicating less significant ones (higher FDR).

Functional enrichment analysis (GO:BP) restricted to these 22 shared differentially abundant proteins revealed a predominant association with biological processes related to cellular metabolism and energy production, including glycolytic process, glucose metabolic process, glucose 6-phosphate metabolic process, and gluconeogenesis (Fig. 7).

At the molecular function level (GO:MF), enrichment analysis identified only a single significantly enriched term, corresponding to small molecule binding (Figure S1), consistent with the predominance of metabolic enzymes within this shared protein set.

Consistently, KEGG pathway enrichment analysis revealed a strong overrepresentation of pathways related to carbohydrate metabolism and energy production, including glycolysis/gluconeogenesis, carbon metabolism, and biosynthesis of amino acids (Figure S2), further highlighting the impact of cryopreservation on metabolic processes supporting ATP production in sperm cells across all sperm origins.

#### Epididymal sperm

**Fig. 8:**
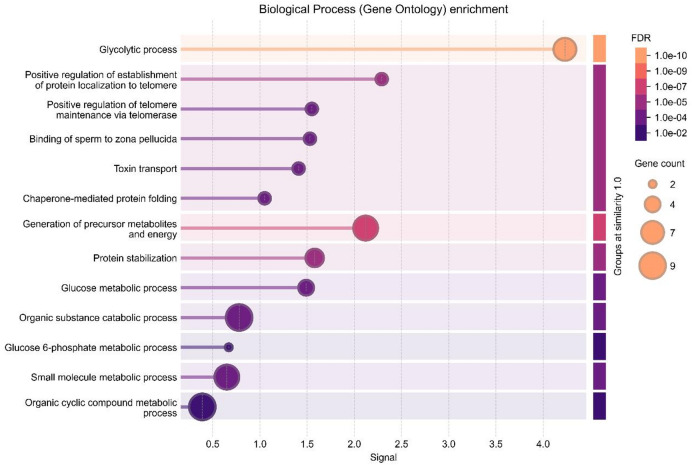
GO:BP enrichment analysis of downregulated proteins in post-thaw epididymal brown bear sperm. Enriched biological processes are ranked by signal strength to emphasize those with the strongest functional relevance. The circle diameter corresponds to the number of proteins mapped to each process, and the color scale denotes the false discovery rate (FDR), with orange indicating more significant enrichments (lower FDR) and purple indicating less significant ones (higher FDR).

Cryopreservation resulted in the significant downregulation of 56 proteins in epididymal sperm with a *q*-value < 0.05 and a |log₂ fold change| ≥ 1. Functional enrichment analysis of these proteins revealed broad alterations in multiple biological processes, including energy metabolism (glycolytic process, glucose metabolic process, glucose 6-phosphate metabolic process, generation of precursor metabolites and energy, organic substance catabolic process, small molecule metabolic process, and organic cyclic compound metabolic process), telomere regulation (positive regulation of establishment of protein localization to telomere and positive regulation of telomere maintenance via telomerase), stress response (toxin transport), protein homeostasis (chaperone-mediated protein folding and protein stabilization), and fertilization-related mechanisms (binding of sperm to zona pellucida) (Fig. 8).

**Fig. 9:**
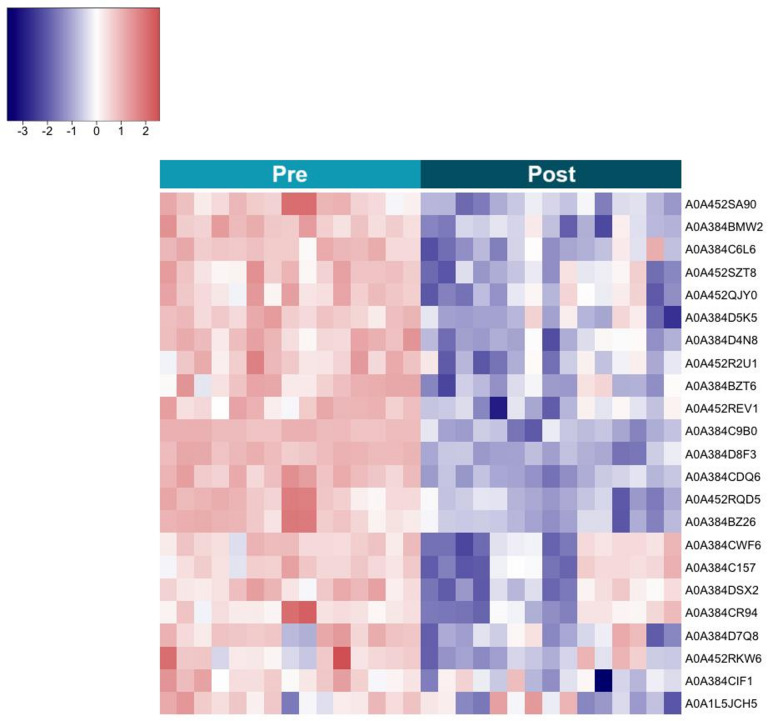
Heatmap showing the impact of cryopreservation on the proteome of brown bear epididymal sperm. Proteins are clustered using hierarchical clustering. Each row represents an individual protein (labelled with its UniProt ID), and each column corresponds to an individual proteomic run (technical replicate) derived from epididymal sperm samples collected from 9 different males (Pre, pre-freeze; Post, post-thaw). The color scale indicates normalized expression levels, with red areas representing higher and blue areas representing lower relative protein abundance. Proteins were normalized and filtered by a |log₂ fold change| ≥ 1 and *q* < 0.05.

To provide a visual overview, a representative subset of these differentially abundant proteins, selected based on consistent detection across all epididymal sperm samples, was used to generate a heatmap illustrating protein abundance changes between pre-freeze and post-thaw conditions (Fig. 9).

#### Pre-ejaculated sperm

**Fig. 10:**
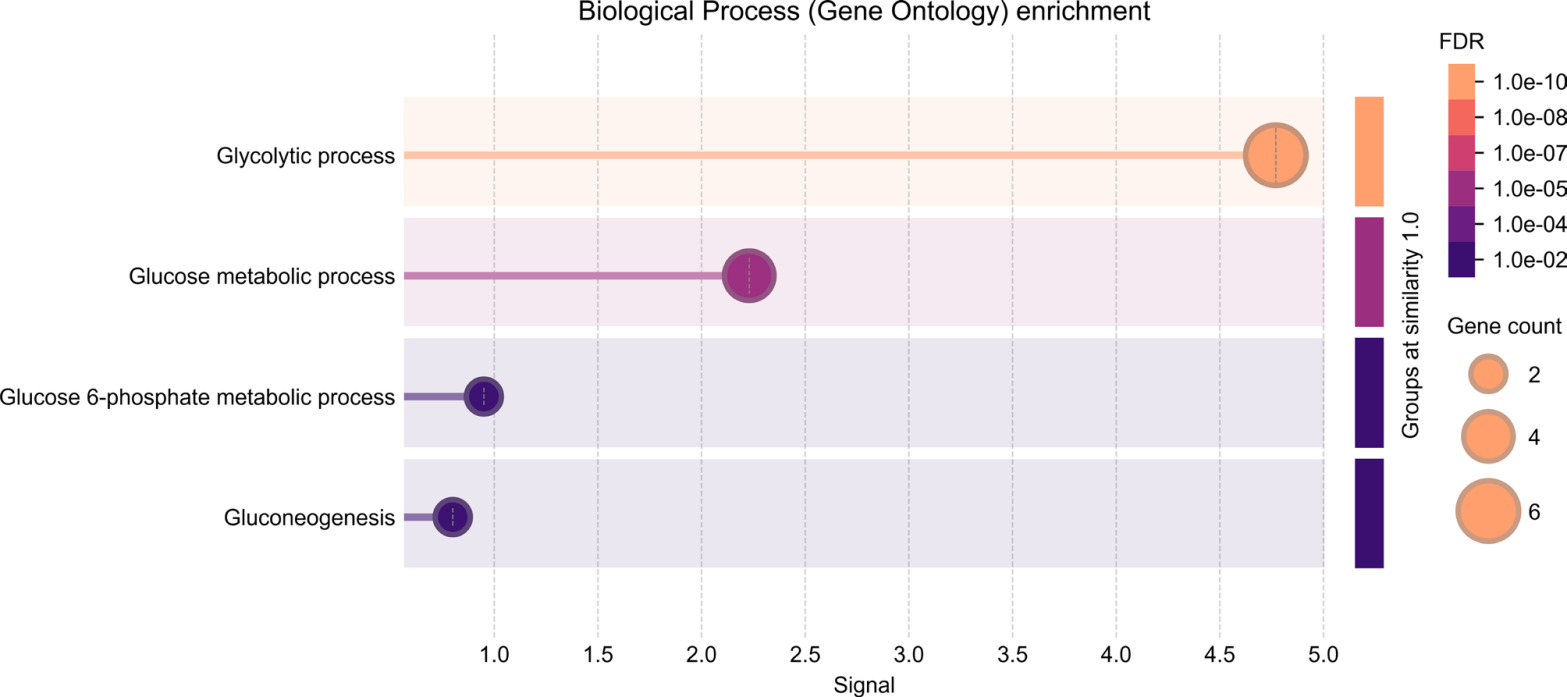
GO:BP enrichment analysis of downregulated proteins in post-thaw pre-ejaculated brown bear sperm. Enriched biological processes are ranked by signal strength to emphasize those with the strongest functional relevance. The circle diameter corresponds to the number of proteins mapped to each process, and the color scale denotes the false discovery rate (FDR), with orange indicating more significant enrichments (lower FDR) and purple indicating less significant ones (higher FDR).

In pre-ejaculated sperm, 33 proteins were significantly downregulated in post-thaw samples compared to pre-freeze samples (*q* < 0.05, |log₂ fold change| ≥ 1). Gene ontology enrichment analysis (GO:BP) of these downregulated proteins revealed a predominant impact on biological processes related to energy metabolism. The most significantly affected GO:BP terms included the glycolytic process, glucose metabolic process, glucose 6-phosphate metabolic process, and gluconeogenesis (Fig. 10), suggesting a disruption of carbohydrate metabolism due to cryoinjury.

**Fig. 11:**
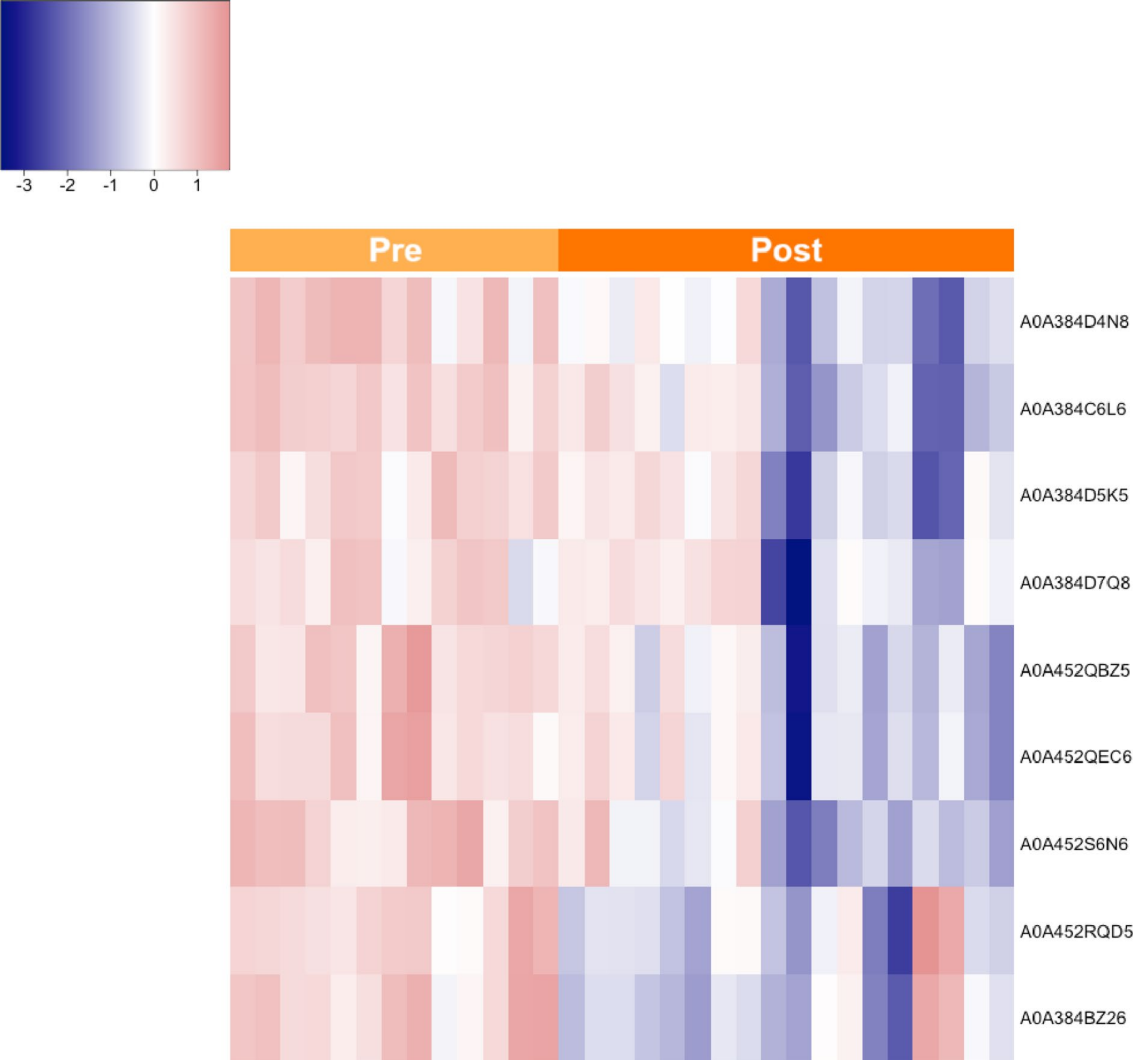
Heatmap showing the impact of cryopreservation on the proteome of brown bear pre-ejaculated sperm. Proteins are clustered using hierarchical clustering. Each row represents an individual protein (labelled with its UniProt ID), and each column corresponds to an individual proteomic run (technical replicate) derived from pre-ejaculated sperm samples collected from 9 different males (Pre, pre-freeze; Post, post-thaw). The color scale indicates normalized expression levels, with red areas representing higher and blue areas representing lower relative protein abundance. Proteins were normalized and filtered by a |log₂ fold change| ≥ 1 and *q* < 0.05.

A heatmap summarizing consistently downregulated proteins in pre-ejaculated sperm is illustrated in Fig. 11.

#### Ejaculated sperm

**Fig. 12:**
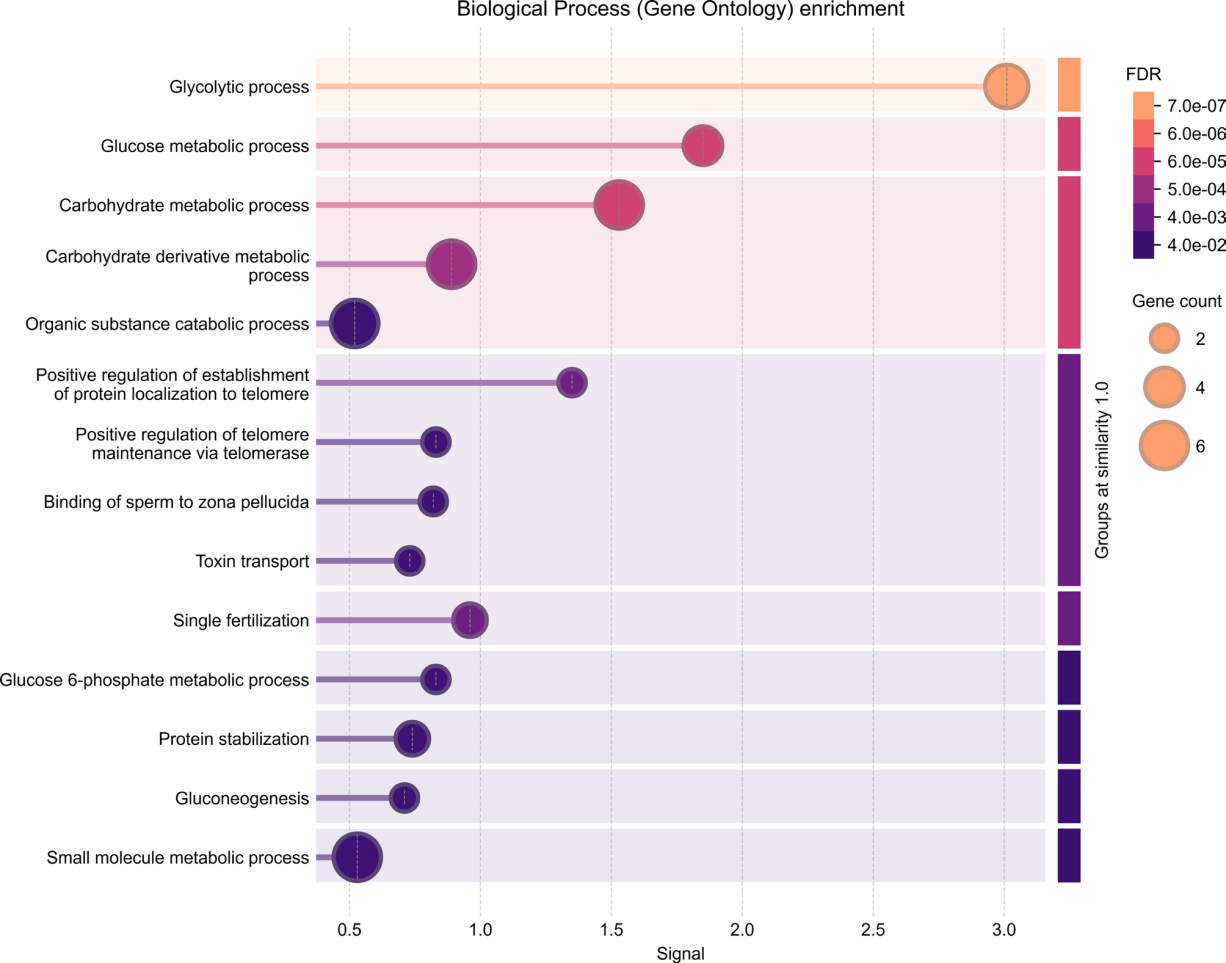
GO:BP enrichment analysis of downregulated proteins in post-thaw ejaculated brown bear sperm. Enriched biological processes are ranked by signal strength to emphasize those with the strongest functional relevance. The circle diameter corresponds to the number of proteins mapped to each process, and the color scale denotes the false discovery rate (FDR), with orange indicating more significant enrichments (lower FDR) and purple indicating less significant ones (higher FDR).

A total of 53 proteins were found to be significantly downregulated in post-thaw samples relative to pre-freeze counterparts (*q* < 0.05, |log₂ fold change| ≥ 1) in ejaculated sperm. GO:BP enrichment analysis of these proteins identified disruptions in a wide range of biological processes, including energy metabolism (glycolytic process, glucose metabolic process, carbohydrate metabolic process, carbohydrate derivate metabolic process, organic substance catabolic process, glucose 6-phosphate metabolic process, gluconeogenesis, and small molecule metabolic process), telomere regulation (positive regulation of establishment of protein localization to telomere and positive regulation of telomere maintenance via telomerase), stress response (toxin transport), protein homeostasis (protein stabilization), and fertilization-related mechanisms (binding of sperm to zona pellucida and single fertilization) (Fig. 12).

**Fig. 13:**
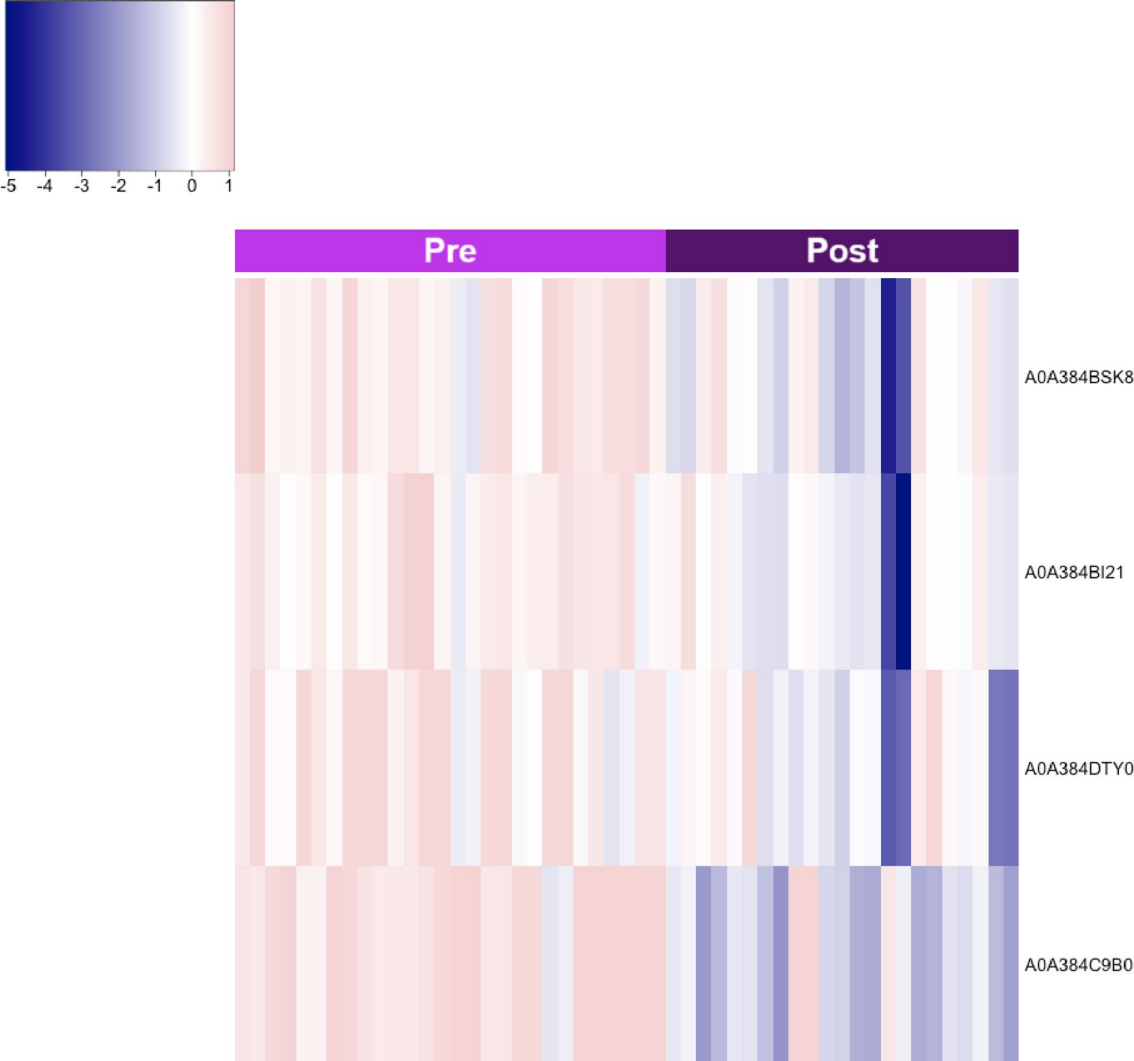
Heatmap showing the impact of cryopreservation on the proteome of brown bear ejaculated sperm. Proteins are clustered using hierarchical clustering. Each row represents an individual protein (labelled with its UniProt ID), and each column corresponds to an individual proteomic run (technical replicate) derived from ejaculated sperm samples collected from 14 different males (Pre, pre-freeze; Post, post-thaw). The color scale indicates normalized expression levels, with red areas representing higher and blue areas representing lower relative protein abundance. Proteins were normalized and filtered by a |log₂ fold change| ≥ 1 and *q* < 0.05.

A representative heatmap was constructed using a reduced subset of significantly downregulated proteins consistently detected in post-thaw ejaculated sperm (Fig. 13).

## Discussion

This study investigated whether sperm collected from epididymal, pre-ejaculatory, and ejaculatory origins in the brown bear (*Ursus arctos*) differ in their ability to withstand cryopreservation through parallel assessments of sperm functionality and proteomic composition in pre- and post-thaw samples. The integration of physiological and molecular data enabled a comprehensive characterization of origin-dependent responses to freeze-thaw stress.

Epididymal sperm displayed the highest pre-freezing values for most motility and kinetic parameters (TM, PM, VCL, and ALH), while viability, apoptosis, and mitochondrial ROS production were comparable among all groups. These results contrast with findings in other species. For instance, in the Iberian red deer, lower CASA parameters were observed in epididymal samples compared to ejaculated ones, despite similar viability^33^. Conversely, García-Álvarez *et al.*^34^ reported in rams that sperm movement characteristics were similar between epididymal and electroejaculated sperm, although viability was higher in epididymal samples. On the other hand, Filliers and co-workers^35^ found poorer motility and morphology in epididymal sperm than in sperm collected by urethral catheterization in domestic cats, whereas Prochowska *et al.*^36^ observed the opposite. These inconsistencies are likely due to differences in sample handling. In both our study and Prochowska’s^36^, epididymal sperm were recovered by epididymal slicing immediately after castration or epididectomy, promptly diluted in an appropriate extender, and thus minimally exposed to adverse conditions. In contrast, other studies involved delayed processing after transport under refrigeration^33^ or at room temperature^34^, or incubation of the sliced epididymis at 39 °C for 20 min to allow passive sperm release into the medium^35^, conditions that could have negatively impacted sperm motility, viability, or morphology.

After cryopreservation, sperm functionality was reduced in all sperm origins (epididymal, pre-ejaculated, and ejaculated), as expected. Among them, ejaculated sperm yielded the most favorable post-thaw profile in terms of viability, apoptotic status, and mitochondrial ROS levels. Although CellROX™ Deep Red does not directly assess mitochondrial membrane potential, this probe has been reported to localize predominantly to the sperm midpiece and to be associated with mitochondria-linked redox processes in ram sperm^37^. In this context, higher CellROX™ Deep Red fluorescence in ejaculated sperm may indicate a more preserved mitochondria-associated redox state after cryopreservation. Nevertheless, this interpretation should be approached with caution, as direct measurements of mitochondrial functionality were not performed in the present study. On the other hand, epididymal sperm exhibited the highest VCL after thawing, although no differences were observed among sperm origins for other sperm motility and kinetic parameters. These findings are somewhat unexpected given that, in most species studied to date, epididymal sperm generally display better cryotolerance than ejaculated sperm. This has been demonstrated in dogs^38^, stallions^39^, bulls^40^, rams^41,42^, and mouflons^43^, where epididymal sperm tend to show better post-thaw motility, viability, structural integrity, and fertilizing ability. Interestingly, in blue wildebeest, electroejaculated sperm exhibited higher post-thaw motility, while epididymal sperm displayed superior viability^44^. By contrast, no differences in post-thaw motility were observed in agoutis between sperm collected by electroejaculation and from the epididymis using retrograde flushing^45^, underscoring the species-specific nature of sperm cryoresistance.

The superior post-thaw performance of ejaculated sperm in brown bears may be attributed to the presence of seminal plasma, which contributes to sperm membrane stabilization through its protein composition^46–48^, amino acid and lipid profiles, antioxidant content^49–51^, and extracellular vesicles^52,53^, thereby buffering damage from cold shock and ice crystal formation. Our oxidation-reduction potential results support this hypothesis. Pre-freeze sORP values were higher in epididymal sperm, indicating a more oxidized baseline status in the absence of seminal plasma. After thawing, sORP increased in ejaculated sperm but remained unchanged in epididymal samples, suggesting that epididymal cells had either already reached a critical oxidative threshold or lacked effective redox buffering mechanisms. Moreover, only ejaculated sperm showed a decrease in cORP, reflecting the consumption of antioxidant reserves during the freeze-thaw process, likely associated with antioxidant components provided by seminal plasma rather than a direct measurement of antioxidant enzymatic activity. Surprisingly, despite the absence of intentional seminal plasma exposure, pre-ejaculated sperm displayed a redox profile more similar to ejaculated than to epididymal sperm. This may reflect the combined influence of residual urethral accessory gland secretions and urinary contamination, as previously reported by our research group in brown bear pre-ejaculated samples^29^, which could modify the oxidative environment and partially mask the expected redox profile. However, neither seminal plasma components nor specific biochemical markers of urine contamination were directly quantified in the present study.

The differential rate (DR) analysis, which normalizes cryoresistance outcomes by accounting for baseline values, further supported these findings. Ejaculated sperm showed higher DR values for both viability and mitochondrial ROS content than epididymal sperm, indicating superior preservation of these parameters relative to their initial state. In contrast, pre-ejaculated sperm exhibited markedly higher DR values for sORP compared to epididymal sperm, reflecting a more pronounced oxidative shift during cryopreservation, possibly due to urinary contaminants exacerbating redox imbalance^54,55^.

While functional analyses revealed the physiological consequences of freezing, proteomic analysis offered insights into the underlying molecular mechanisms. However, some methodological considerations should be taken into account when interpreting the proteomic results. Changes in protein abundance after cryopreservation do not necessarily reflect active biological regulation and could also arise from structural effects such as membrane destabilization, protein delocalization, loss of loosely associated cytoplasmic or surface-bound proteins, or differences in extraction efficiency between pre-freeze and post-thaw samples, as reported in other studies^56–58^. These effects may be more pronounced in epididymal and, to a lesser extent, in pre-ejaculated sperm, which are either not exposed or only minimally exposed to the stabilizing influence of seminal plasma. Moreover, proteomic analyses in non-model wildlife species are constrained by annotation limitations. In this study, protein identification and functional annotation relied on the UniProt/Bear database, which includes entries from closely related ursid species (mainly *Ursus americanus* and *Ursus maritimus*)^59,60^, and therefore functional interpretations should be viewed within a comparative ursid framework. It should be noted that, due to the use of orthologous databases, multiple UniProt entries corresponding to the same protein were detected and treated as independent features, which may account for apparently divergent regulation patterns among related entries, including the detection of different orthologous entries of the same protein among both up- and downregulated proteins. In such cases, opposite trends are more likely to reflect isoform-specific responses, partial sequence coverage, or database redundancy and peptide-to-protein assignment issues, rather than true biological contradictions in the regulation of a single protein.

Within this methodological context, the overlap analysis of downregulated proteins across sperm origins provides an overview of shared and origin-specific proteomic responses to cryopreservation. Notably, the presence of a common subset of 22 consistently downregulated proteins defines a conserved molecular signature of cryodamage, mainly associated with core metabolic functions and energy production. In parallel, origin-specific proteins support distinct cryopreservation-associated proteomic patterns depending on sperm source. These shared and divergent signatures are discussed below in relation to the functional and enrichment results observed within each origin.

Epididymal sperm exhibited the most pronounced proteomic response to cryopreservation, with 56 downregulated proteins and 13 significantly affected biological processes, as revealed by GO:BP enrichment. Many of these processes were related to energy metabolism, with the reduced abundance of key enzymes such as Phosphoglycerate mutase (PGAM), Phosphoglycerate kinase (PGK), and Glyceraldehyde-3-phosphate dehydrogenase (GAPDH). These enzymes are essential for ATP generation through glycolysis, which sustains sperm motility and fertilization ability^61–66^. PGAM, in particular, has been identified as one of the most abundant proteins in epididymal sperm from wild ruminants such as ibex, mouflon, and chamois^25^, suggesting a potential contribution to high sperm cryotolerance^67^. While this observation supports the functional relevance of PGAM, further studies are needed to assess whether variations in its expression influence sperm freezability. In the brown bear, the marked downregulation of PGAM after thawing, alongside other glycolytic enzymes, may contribute to the post-thaw decline in motility observed in epididymal sperm, consistent with findings in Addra gazelle ejaculated sperm^68^.

Beyond energy metabolism, epididymal sperm showed enrichment of GO:BP terms related to telomere regulation, including positive regulation of establishment of protein localization to telomere and telomere maintenance via telomerase. These biological processes are crucial for maintaining genomic integrity and chromatin organization during gametogenesis^69^. Although telomere dynamics are not commonly assessed in the context of sperm cryobiology, recent evidence links telomere alterations in length and function with oxidative stress and activation of apoptosis pathways in sperm^70^, with potential consequences on sperm quality^71–74^, fertility^75,76^, and embryo development^77,78^ in humans and boars. Therefore, the downregulation of telomere-associated proteins observed in brown bear post-thaw epididymal samples may be associated with molecular patterns previously linked to genomic instability under oxidative stress conditions. However, no direct assessment of DNA integrity was performed in this study.

Cryopreservation also appeared to compromise stress response mechanisms in epididymal sperm through the downregulation of proteins like Sodium/potassium-transporting ATPase subunit alpha, which is key for maintaining ion homeostasis and membrane potential, as well as sperm motility and function^79–81^; Glutathione S-transferase (GST), an essential antioxidant enzyme involved in detoxification of ROS, preserving sperm plasma membrane stability and mitochondrial function^82^, which has been established as a fertility^83^ and cryotolerance^84^ biomarker in boar sperm; and Clusterin (CLU), a multifunctional protein with antioxidant properties that protects bovine sperm against oxidative stress-induced apoptosis, thereby contributing to sperm longevity^85,86^. The reduced abundance of all these proteins suggests an impaired antioxidant defense and increased susceptibility to oxidative damage in post-thaw brown bear epididymal sperm.

Proteostasis mechanisms were also affected in epididymal sperm, as indicated by the downregulation of biological processes related to protein folding, stabilization, and trafficking. These alterations may impair the ability of sperm cells to maintain protein conformation and function under freezing conditions. Notably, members of the Heat Shock 70 kDa family (HSP70) were among the affected proteins. HSP70 acts as a molecular chaperone, facilitating protein folding and assembly, stabilizing partially unfolded proteins, and preventing protein aggregation^87^, while also protecting cells from the effects of oxidative stress and apoptosis^88^. Several studies have demonstrated that reduced levels of HSP70 are associated with decreased sperm quality after cooling or freezing and thawing in species such as buffalo^89,90^, bulls^23,91^, and pigs^92,93^. Similarly, there is evidence that CLU acts as a chaperone in human seminal plasma^94^ and is involved in sperm maturation in bulls^95^, mice^96^, and humans^97,98^. Therefore, the decreased abundance of these chaperone proteins in frozen-thawed brown bear epididymal sperm may compromise their resilience to cold shock, increasing susceptibility to structural damage and apoptotic processes.

In parallel, one fertilization-related biological process, the binding of sperm to zona pellucida, was significantly downregulated following cryopreservation in epididymal sperm. Among the affected proteins was A-kinase anchor protein 3 (AKAP3), a critical component of the sperm fibrous sheath involved in organizing protein kinase A (PKA) signalling, supporting flagellar integrity and sperm motility^99–102^, and orchestrating capacitation through its controlled proteasomal degradation during sperm maturation^103–105^. Reduced expression of AKAP3 has been associated with impaired sperm morphology and asthenoteratozoospermia in humans^106^ and mice^107^. Additionally, two actin isoforms (Actin alpha 2, smooth muscle, and Actin gamma 1) were also downregulated. Actin is a key cytoskeletal component in sperm, playing a crucial role in maintaining acrosomal architecture and facilitating the acrosome reaction^108^. In bovine sperm, actin is enriched in the acrosomal and postacrosomal regions and undergoes dynamic reorganization during capacitation and acrosomal exocytosis^109,110^. Altogether, the observed downregulation of AKAP3 and actin isoforms in brown bear post-thaw epididymal sperm suggests molecular alterations in proteins involved in sperm function and oocyte recognition, which could potentially influence fertilization-related processes. Indeed, cryopreservation is known to disrupt membrane architecture and protein localization in sperm, impairing acrosomal responsiveness and fertilization efficiency in stallions^111^, dogs^112^, and humans^113^. Thus, the observed decrease in zona pellucida-binding proteins may compromise the ability of brown bear epididymal sperm to complete the fertilization process after thawing. However, fertilization competence was not directly evaluated in the present study.

Pre-ejaculated sperm exhibited the mildest proteomic response to cryopreservation, with only 33 downregulated proteins and four affected biological processes, all of which were related to energy metabolism. The heatmap for this group highlighted key glycolytic enzymes, including PGAM, PGK, and Beta-enolase. Specifically, Beta-enolase is localized in the sperm flagellum, where it supports ATP generation for motility and fertility^114^ and interacts with microtubules to maintain structural integrity of the sperm tail^115^. Therefore, these alterations may reflect a loss of energetic competence post-thaw, consistent with the moderate decline in motility and mitochondrial ROS production observed in this group. Although no enriched biological processes related to stress response or fertilization were detected in pre-ejaculated sperm, several individual proteins involved in these processes were also downregulated, including GST, AKAP3, Actin alpha 2, smooth muscle, and Actin gamma 1 (Table S2). The downregulation of these proteins, despite the lack of enrichment in their associated biological processes, may suggest subtle molecular disruptions that could still impact sperm function after thawing, particularly at the levels of the antioxidant defense system, acrosome integrity, and zona pellucida binding process.

Finally, ejaculated sperm appeared to undergo a proteomic response to cryopreservation similar to that of epididymal sperm, with 53 proteins downregulated and 14 enriched biological processes, including those associated with energy metabolism, telomere regulation, protein homeostasis, and fertilization. Nevertheless, the heatmap featured only four proteins, among which were GAPDH, L-lactate dehydrogenase (LDH), and ATP synthase subunit beta (ATP5F1B). Particularly noteworthy is LDH, which in sperm cells exists predominantly as the testis-specific isoenzyme LDH-X or C_4_^116,117^. This isoform is uniquely adapted to meet the energetic demands of sperm and is primarily localized in the midpiece and principal piece of the flagellum, playing a central role in maintaining the ATP levels required for motility^118–120^, especially under anaerobic or stressful conditions^121,122^. The downregulation of LDH observed post-thaw may therefore reflect a reduced metabolic flexibility and a decreased capacity to buffer energy needs after cryoinjury. Interestingly, studies in rodents have shown that LDH-X activity declines during epididymal transit, indicating its potential modulation during sperm maturation and its sensitivity to environmental or physiological changes^123^. Likewise, the decreased abundance of ATP5F1B, a catalytic subunit of mitochondrial ATP synthase, may compromise oxidative phosphorylation efficiency, further contributing to sperm motility suppression^124^. Its role extends beyond energy production, as it has also been shown to be essential for germ cell maturation and male fertility, as demonstrated in model organisms such as *Drosophila*^125^. In addition to energy metabolism, Albumin (ALB), a multifunctional protein from testicular, epididymal, and prostatic origins^126,127^, was also downregulated in ejaculated sperm following cryopreservation. Beyond its well-established role in sperm capacitation (through cholesterol extraction and modulation of membrane fluidity)^128–131^, ALB exerts potent antioxidant and cryoprotective functions. It scavenges ROS and protects membrane lipids from peroxidation^132,133^, which is particularly relevant during freezing and thawing procedures. Moreover, its abundance has been positively correlated with cryotolerance in boar sperm, highlighting its potential role as a biomarker of freezability^134^. Therefore, the reduced presence of albumin post-thaw may compromise the sperm’s ability to counteract oxidative stress, ultimately affecting membrane stability and post-thaw viability. Together, the underrepresentation of these proteins reinforces the idea that energy production and stress response biological processes are particularly vulnerable to cryodamage, even in ejaculated sperm.

In summary, the integration of functional and proteomic data across the three sperm origins reveals distinct post-thaw quality profiles that reflect differential cryotolerance among brown bear sperm sources. Epididymal sperm, despite displaying superior pre-freeze motility and kinetic values, experienced the most extensive proteomic disruptions after cryopreservation, particularly in biological processes related to energy metabolism, stress response, protein homeostasis, and fertilization-related processes. Pre-ejaculated sperm showed a more moderate response, with proteomic alterations primarily confined to glycolytic metabolism, suggesting a more localized cryoinjury. Ejaculated sperm, while also exhibiting significant changes in energy metabolism and antioxidant defenses, retained the highest post-thaw viability and mitochondrial ROS production, likely due to the protective effects of seminal plasma. This work provides novel insights into the differential response of sperm origins to freezing and highlights key biological processes associated with cryodamage, with potential implications for wildlife conservation and gamete biobanking strategies.

## Supplementary Information

Below is the link to the electronic supplementary material.

Supplementary Material 1

Supplementary Material 2

Supplementary Material 3

Supplementary Material 4

## Data Availability

All quantitative proteomic data supporting the findings of this study are provided as Supplementary Data S1-S3 . In addition, the complete protein abundance dataset, including protein-level quantification for individual samples, has been deposited in the institutional repository of Universidad de León and is publicly available at https://hdl.handle.net/10612/26988.
