# Supplementary material for: Cryotolerance of brown bear (Ursus arctos) sperm depends on sperm origin: insights into sperm quality and proteomic profiles
Source: Sci Rep. 2026 May 8;16:21128. doi: 10.1038/s41598-026-43295-0 (PMC13342079; doi:10.1038/s41598-026-43295-0)
Supplement: Supplementary file 4 — Supplementary Material 4 [file 41598_2026_43295_MOESM4_ESM.pdf]

SUPPLEMENTARY INFORMATION

Table S1. List of the 22 proteins consistently downregulated after cryopreservation across all sperm origins in brown bear sperm.

UniProt ID	Protein	Organism
A0A384D7Q8	A-kinase anchor protein 3	Polar bear (*Ursus maritimus*)
A0A452RKW6	Hexokinase	American black bear (*Ursus americanus*)
A0A384CIF1		Polar bear (*Ursus maritimus*)
A0A452QEC6	Beta-enolase	American black bear (*Ursus americanus*)
A0A452QBZ5		
A0A384D4N8	Phosphoglycerate mutase	Polar bear (*Ursus maritimus*)
A0A452S6N6	Phosphoglycerate kinase	American black bear (*Ursus americanus*)
A0A384C6L6		Polar bear (*Ursus maritimus*)
A0A384BTP3	60 kDa Heat shock protein, mitochondrial	Polar bear (*Ursus maritimus*)
A0A452RRQ9	Glucose-6-phosphate isomerase	American black bear (*Ursus americanus*)
A0A384BJG3	Heat shock protein 90 alpha family class A member 1	Polar bear (*Ursus maritimus*)
A0A452REV1	T-complex protein 1 subunit gamma	American black bear (*Ursus americanus*)
A0A384BZT6	T-complex protein 1 subunit epsilon	Polar bear (*Ursus maritimus*)
A0A452QJY0	Sodium/potassium-transporting ATPase subunit alpha	American black bear (*Ursus americanus*)
A0A452SZT8		Polar bear (*Ursus maritimus*)
A0A384CDQ6	Parkinson disease protein 7 homolog	Polar bear (*Ursus maritimus*)
A0A452SA90	Glyceraldehyde-3-phosphate dehydrogenase	American black bear (*Ursus americanus*)
A0A8M1GQR3	Triosephosphate isomerase	Polar bear (*Ursus maritimus*)
A0A452RQD5	Actin gamma 1	American black bear (*Ursus americanus*)
A0A384BZ26	Actin alpha 2, smooth muscle	Polar bear (*Ursus maritimus*)
A0A384C9B0	Albumin	Polar bear (*Ursus maritimus*)
A0A384D8F3	Clusterin	Polar bear (*Ursus maritimus*)

UniProt IDs, protein names, and annotated species for the 22 proteins consistently downregulated after cryopreservation in epididymal, pre-ejaculated, and ejaculated brown bear sperm ($q < 0.05$, $|\log_2 \text{fold change}| \geq 1$). Protein identification was based on orthologous entries from related *Ursus* species due to the lack of a fully curated *Ursus arctos* proteome.

Supplementary Data S1. Differential proteomic analysis of epididymal brown bear sperm before and after cryopreservation. This Excel file contains the results of the differential protein enrichment analysis comparing pre-freeze and post-thaw epididymal sperm samples. The worksheet "DEPs" lists proteins with significant differential abundance after cryopreservation, filtered using a threshold of $|\log_2$ fold change$| \geq 1$ and q-value ≤ 0.05. Protein IDs highlighted in red indicate proteins with higher relative abundance in post-thaw samples, whereas those highlighted in blue indicate proteins with lower relative abundance after cryopreservation. The worksheet "All" contains the complete list of proteins quantified across the study after quality filtering, which constitutes the reference dataset used for the differential analyses.

Supplementary Data S2. Differential proteomic analysis of pre-ejaculated brown bear sperm before and after cryopreservation. This Excel file contains the results of the differential protein enrichment analysis comparing pre-freeze and post-thaw pre-ejaculated sperm samples. The worksheet "DEPs" lists proteins with significant differential abundance after cryopreservation, filtered using a threshold of $|\log_2$ fold change$| \geq 1$ and q-value ≤ 0.05. Protein IDs highlighted in red indicate proteins with higher relative abundance in post-thaw samples, whereas those highlighted in blue indicate proteins with lower relative abundance after cryopreservation. The worksheet "All" contains the complete list of proteins quantified across the study after quality filtering, which constitutes the reference dataset used for the differential analyses.

Supplementary Data S3. Differential proteomic analysis of ejaculated brown bear sperm before and after cryopreservation. This Excel file contains the results of the differential protein enrichment analysis comparing pre-freeze and post-thaw ejaculated sperm samples. The worksheet "DEPs" lists proteins with significant differential abundance after cryopreservation, filtered using a threshold of $|\log_2$ fold change$| \geq 1$ and q-value ≤ 0.05. Protein IDs highlighted in red indicate proteins with higher relative abundance in post-thaw samples, whereas those highlighted in blue indicate proteins with lower relative abundance after cryopreservation. The worksheet "All" contains the complete list of proteins quantified across the study after quality filtering, which constitutes the reference dataset used for the differential analyses.

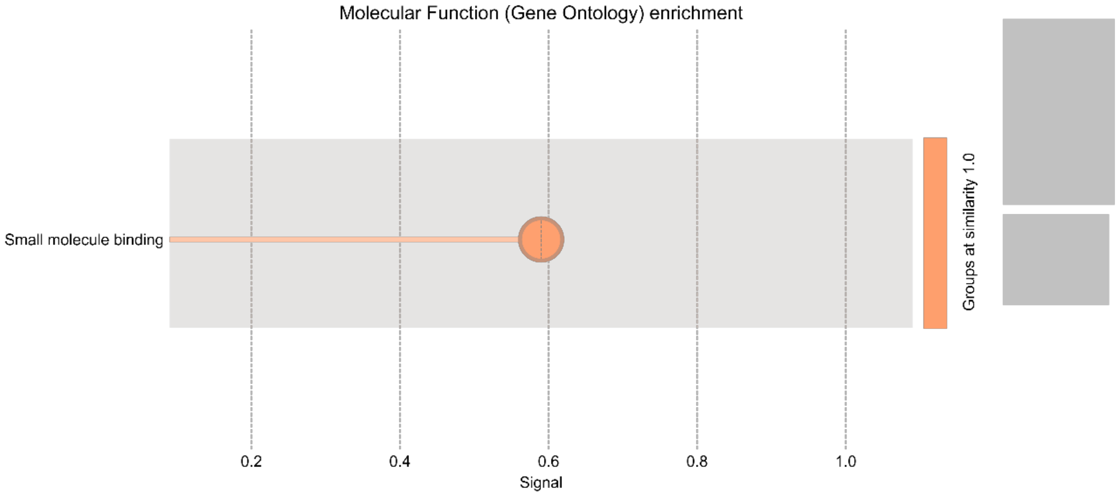

Fig. S1. GO:MF enrichment analysis of the 22 proteins consistently downregulated after cryopreservation across all sperm origins in brown bear sperm. The circle diameter corresponds to the number of proteins mapped to the enriched molecular function.

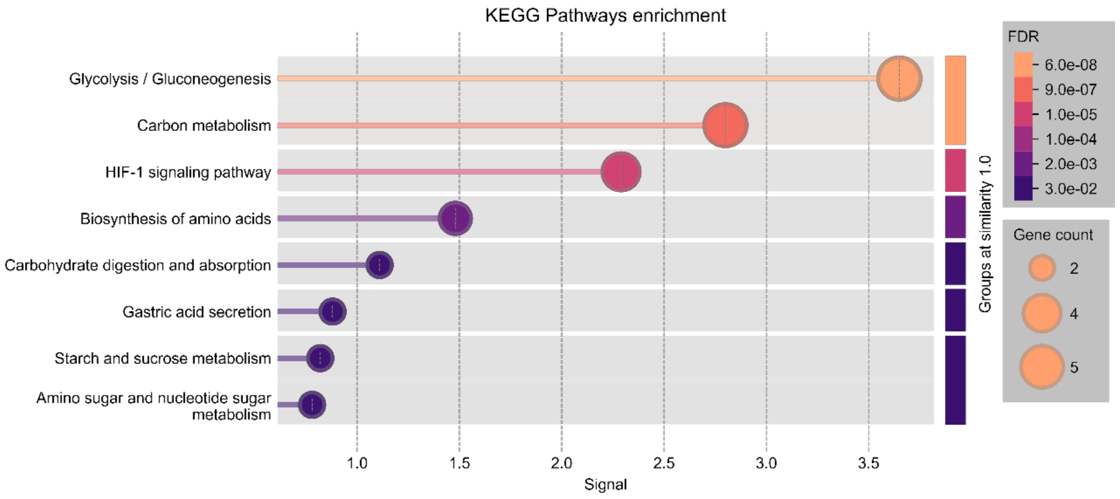

Fig. S2. KEGG pathway enrichment analysis of the 22 proteins consistently downregulated after cryopreservation across all sperm origins in brown bear sperm. Enriched pathways are ranked by signal strength to emphasize those with the strongest functional relevance. The circle diameter corresponds to the number of proteins mapped to each pathway, and the color scale denotes the false discovery rate (FDR), with orange indicating more significant enrichments (lower FDR) and purple indicating less significant ones (higher FDR).

Table S2. Functional classification of proteins consistently downregulated after cryopreservation.

Functional category	UniProt ID	Protein	Organism	EP	PR	EJ
**Energy metabolism**	A0A384BMW2	Fructose-bisphosphate aldolase	Polar bear (*Ursus maritimus*)	✓		
	A0A452RKW6<br>A0A384CIF1	Hexokinase	American black bear (*Ursus americanus*)<br>Polar bear (*Ursus maritimus*)	✓		
	A0A452R2U1<br>A0A384D4N8	Phosphoglycerate mutase	American black bear (*Ursus americanus*)<br>Polar bear (*Ursus maritimus*)	✓	✓	
	A0A452S6N6<br>A0A384C6L6	Phosphoglycerate kinase	American black bear (*Ursus americanus*)<br>Polar bear (*Ursus maritimus*)	✓	✓	
	A0A452QEC6<br>A0A452QBZ5	Beta-enolase	American black bear (*Ursus americanus*)		✓	
	A0A452SA90	Glyceraldehyde-3-phosphate dehydrogenase	American black bear (*Ursus americanus*)	✓		✓
	A0A384BSK8	L-lactate dehydrogenase	Polar bear (*Ursus maritimus*)			✓
	A0A384BI21	ATP synthase subunit beta	Polar bear (*Ursus maritimus*)			✓
**Stress response**	A0A452QJY0<br>A0A452SZT8	Sodium/potassium-transporting ATPase subunit alpha	American black bear (*Ursus americanus*)<br>Polar bear (*Ursus maritimus*)	✓		
	A0A384D5K5	Glutathione S-transferase	Polar bear (*Ursus maritimus*)	✓	✓	
	A0A384D8F3	Clusterin	Polar bear (*Ursus maritimus*)	✓		
	A0A384C9B0	Albumin	Polar bear (*Ursus maritimus*)	✓		✓
**Protein homeostasis**	A0A384BZT6	T-complex protein 1 subunit epsilon	Polar bear (*Ursus maritimus*)	✓		
	A0A452REV1	T-complex protein 1 subunit gamma	American black bear (*Ursus americanus*)	✓		
	A0A384CWF6	Heat shock-related 70 kDa protein 2	Polar bear (*Ursus maritimus*)	✓		
	A0A384C157	Heat shock cognate 71 kDa protein	Polar bear (*Ursus maritimus*)	✓		
	A0A384DSX2	Heat shock 70 kDa protein 1-like	Polar bear (*Ursus maritimus*)	✓		
	A0A384CR94	Endoplasmic reticulum chaperone BIP	Polar bear (*Ursus maritimus*)	✓		
**Fertilization-related mechanisms**	A0A384D7Q8	A-kinase anchor protein 3	Polar bear (*Ursus maritimus*)	✓	✓	
	A0A384BZ26	Actin alpha 2, smooth muscle	Polar bear (*Ursus maritimus*)	✓	✓	
	A0A452RQD5	Actin gamma 1	American black bear (*Ursus americanus*)	✓	✓	

List of proteins significantly downregulated in post-thaw epididymal (EP), pre-ejaculated (PR), and ejaculated (EJ) brown bear sperm ($q < 0.05$, $|\log_2 \text{fold change}| \geq 1$), grouped into major functional categories based on GO:BP results. These categories (energy metabolism, stress

response, protein homeostasis, and fertilization-related functions) were defined by clustering related terms from enrichment analyses. Check marks indicate the sperm origin in which each protein was identified as downregulated. Overlaps reflect proteins shared across sperm origins and/or annotated in different ursid species.
